# Evaluation of treadmill exercise effect on muscular lipid profiles of diabetic fatty rats by nanoflow liquid chromatography–tandem mass spectrometry

**DOI:** 10.1038/srep29617

**Published:** 2016-07-08

**Authors:** Jong Cheol Lee, Il Yong Kim, Yeri Son, Seul Kee Byeon, Dong Hyun Yoon, Jun Seok Son, Han Sol Song, Wook Song, Je Kyung Seong, Myeong Hee Moon

**Affiliations:** 1 Department of Chemistry, Yonsei University, Seoul, 03722, Korea; 2 College of Veterinary Medicine, Seoul National University, Seoul, 08826, Korea; 3 Korea Mouse Phenotyping Center (KMPC), Seoul National University, Seoul, 08826, Korea; 4 Health and Exercise Science Laboratory, Institute of Sport Science, Seoul National University College of Education, Seoul, 08826, Korea; 5 Institute on Aging, Seoul National University College of Medicine, Seoul, 08826, Korea

## Abstract

We compare comprehensive quantitative profiling of lipids at the molecular level from skeletal muscle tissues (gastrocnemius and soleus) of Zucker diabetic fatty rats and Zucker lean control rats during treadmill exercise by nanoflow liquid chromatography–tandem mass spectrometry. Because type II diabetes is caused by decreased insulin sensitivity due to excess lipids accumulated in skeletal muscle tissue, lipidomic analysis of muscle tissues under treadmill exercise can help unveil the mechanism of lipid-associated insulin resistance. In total, 314 lipid species, including phospholipids, sphingolipids, ceramides, diacylglycerols (DAGs), and triacylglycerols (TAGs), were analyzed to examine diabetes-related lipid species and responses to treadmill exercise. Most lysophospholipid levels increased with diabetes. While DAG levels (10 from the gastrocnemius and 13 from the soleus) were >3-fold higher in diabetic rats, levels of most of these decreased after exercise in soleus but not in gastrocnemius. Levels of 5 highly abundant TAGs (52:1 and 54:3 in the gastrocnemius and 48:2, 50:2, and 52:4 in the soleus) displaying 2-fold increases in diabetic rats decreased after exercise in the soleus but not in the gastrocnemius in most cases. Thus, aerobic exercise has a stronger influence on lipid levels in the soleus than in the gastrocnemius in type 2 diabetic rats.

Lipids are major components of the cell membrane and are involved in energy storage, signal transduction across cell membranes, cell growth, and apoptosis^1^,^2^. Lipidomic analysis is of increasing interest in studying the relationship between the role of lipids and pathogenesis of several diseases such as diabetes, obesity, cardiovascular diseases, and cancers^3^,^4^,^5^. Diabetes mellitus (DM), commonly known as diabetes, is a metabolic disease caused by insulin deficiency (type I: insulin-dependent DM) or malfunctioning (type II: insulin resistance) in which cells fail to respond to insulin, resulting in the blockage of glucose uptake by cells. As type II diabetes is the most common form (90–95%) of DM^6^ and is considered to occur from obesity and lack of physical exercise, numerous studies are being conducted to prevent and treat type II DM by examining the influence of proper exercise, control of body weight, and healthy diets. In particular, excess lipid accumulation in nonadipose tissue such as the heart, liver, kidneys, and skeletal muscle causes lipotoxicity, which induces cell dysfunction and death, leading to type II DM^7^,^8^.

In particular, skeletal muscle is responsible for the major portion of insulin-stimulated whole body glucose disposal and hence it plays important roles in the pathogenesis of insulin resistance. An acute increase in plasma free fatty acids (FFAs), via intravenous lipid infusion, can induce skeletal muscle insulin resistance in nondiabetic and diabetic subjects^9^, whereas an acute decrease in elevated plasma FFA levels lowers insulin resistance in obese diabetic and nondiabetic subjects^10^. Insulin sensitivity is decreased in insulin resistance models when excess lipids are accumulated in skeletal muscles in the form of lipid droplets called intramyocellular lipids (IMCLs)^11^,^12^, and the level of triacylglycerol (TAG), the core species of IMCLs, increases extraordinarily with type II DM^13^. In a type II diabetic rat model, increased muscular TAG level is correlated with reduced activity of insulin-stimulated glycogen synthase, which has a negative effect on insulin sensitivity^14^,^15^. However, the relationship between the amount of intramuscular TAG and insulin resistance is not fully proven. There is a correlation between diacylglycerol (DAG) levels and insulin resistance after high-fat diet feeding in Zucker diabetic fatty (ZDF) rats^16^,^17^. Because DAG is a second intracellular messenger, and because it can activate protein kinase C (PKC), it is thought to negatively affect insulin signaling^18^. Although recent studies uncovered that ceramide (Cer) levels increase in the insulin-resistant muscle of Zucker rats^19^ and that insulin sensitivity can be influenced by an increase in lipotoxic lipid intermediates such as DAG and Cer, known as cell signaling lipids^20^,^21^, whether DAG and Cer levels directly influence insulin resistance remains unclear^22^. These studies focused on examining a certain type of lipids because comprehensive analysis of lipids remains complicated due to the complexity of molecular structures of lipids and their chemical natures.

Lipid analysis has been accelerated by sophisticated electrospray ionization–mass spectrometry (ESI–MS) methods that provide high-speed analysis of lipid molecules with structural determination from fragment ion patterns utilizing tandem MS analysis. Use of liquid chromatography (LC) with MS expands its analytical power by separating complicated lipid mixtures in their intact states, minimizing ion-suppressing effects from highly abundant species^23^,^24^. Recently, nanoflow LC–ESI–MS/MS has been utilized for quantitative analysis of plasma and urinary lipids in patients with prostate cancer, breast cancer, Gaucher disease, and cardiovascular diseases with its low femtomolar detection limit^25^,^26^,^27^,^28^,^29^,^30^.

In this study, comprehensive quantitative profiling of lipids at molecular levels was attempted to examine changes in lipid levels in skeletal muscles of diabetic rats [type II ZDF rats were compared with Zucker lean control (ZLC) rats] under treadmill exercise as a factor of diabetic pathogenesis using nLC–ESI–MS/MS (Fig. 1). Because insulin resistance develops at 7–8 weeks of age^31^, 8-week-old ZDF and ZLC rats were subjected to treadmill exercise for 6 weeks after 1 week of adapted activities, and 2 types of skeletal muscle tissues, the gastrocnemius (muscle fibers primarily involved in running or fast movements of leg) and soleus (primary active muscle to aid stand still), of rats were examined before and after treadmill exercise. In total, 314 lipid species, including various phospholipids (PLs), sphingolipids (SLs), Cers, DAGs, and TAGs, in both muscle tissues were characterized via collision-induced dissociation (CID) experiments by nLC–ESI–MS/MS. Identified lipids were quantified using nanoflow ultrahigh performance LC (nUPLC) with triple quadrupole MS based on the selected reaction monitoring (SRM) method. Changes in levels of individual lipid species were statistically examined to identify lipid species closely related to diabetes and assess the effect of treadmill exercise on lipid profiles of both skeletal muscles in animals with type II diabetes.

## Results

### Effect of treadmill exercise on body and skeletal tissue weights and glucose levels

Body weights of all rats during week 9 to week 15 were plotted in Supplementary Fig. S1 as the cumulative weight gain, revealing gradual increases among all groups, but a steeper increase was observed in ZDF groups {Diabetic control (D) and Diabetic control with exercise (DX)} than in ZLC groups {Lean control (C) and Lean control with exercise (CX)}. After training, the CX group displayed a significant difference in the final body weight, which did not increase as much as that in the C group. The final body weight was not significantly different between DX and D groups. As shown in Supplementary Table S1, the effect of treadmill exercise on losses of body weight, epididymal fat, and gastrocnemius and soleus weights was relatively small. By counting changes in comparison to body weight after exercise, no differences in muscle weight were observed, excluding the increase in the soleus weight in the DX group. The ratio of skeletal muscle weight to body weight in ZDF rats was lower than that in ZLC rats. This is similar to the result of previous studies^32^ because metabolic dysfunction, similar to insulin resistance, results in failure of the physiological growth of muscle. The fasted blood glucose level after training was attenuated in DX and CX groups (Supplementary Table S1).

### Qualitative and quantitative analysis of lipids from muscle tissue samples

Molecular structures of lipids from muscle tissue samples were characterized first via non-targeted global searching by nLC–ESI–MS/MS, and the analysis was performed in positive and negative ion modes of MS for each sample because some species (DAG and TAG) can only be detected in the positive ion mode. Supplementary Fig. S2 shows the base peak chromatograms of a mixture of 28 standard lipids obtained in the positive and negative ion modes, and the same run conditions were applied for the structural identification of muscle tissue lipids throughout the analysis. Lipid molecular structures in subclasses of lysophosphatidylcholine (LPC), phosphatidylcholine (PC), lysophosphatidylethanolamine (LPE), phosphatidylethanolamine (PE), sphingomyelin (SM), DAG, and TAG were identified from CID spectra obtained in the positive ion mode and those of lysophosphatidylglycerol (LPG), phosphatidylglycerol (PG), lysophosphatidylinositol (LPI), phosphatidylinositol (PI), lysophosphatidic acid (LPA), phosphatidic acid (PA), lysophosphatidylserine (LPS), phosphatidylserine (PS), Cer, monohexosylceramide (MHC), dihexosylceramide (DHC), and cardiolipin (CL) were obtained in the negative ion mode. While molecular structures of PLs can be clearly obtained with the position and length of acyl chains together with the number of double bonds from data-dependent CID spectra, the exact location of acyl chains for neutral lipids such as DAG and TAG cannot be distinguished because structural isomers, including regioisomers, are not completely resolved by LC separation. For instance, CID spectra in Supplementary Fig. S3 represent the MS/MS spectra of structural isomers of a) 52:3-TAG (m/z 874.7, [M + NH_4_]^+^, t_r_ = 60.86 min) and b) 36:2-DAG (m/z 638.5, [M + NH_4_]^+^, t_r_ = 49.57 min) obtained from nLC–ESI–MS/MS analysis of a pooled soleus lipid sample in the D group, expressed with the total chain length and number of double bonds. Details of structural determination of DAG and TAG are provided in Supplementary Information.

### Lipid profiles in diabetic and control rats

**Figure 2:**
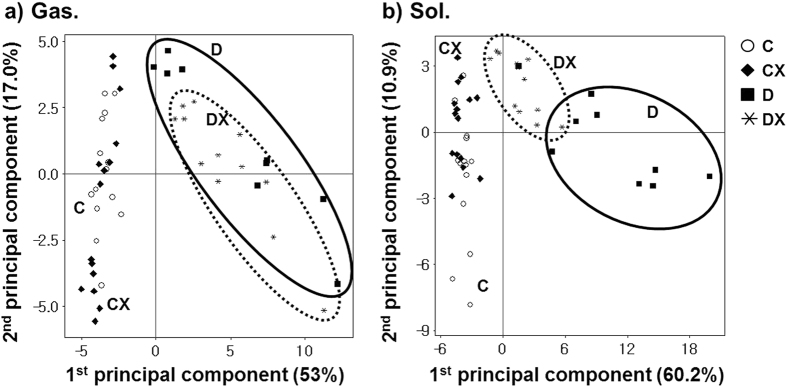
Score plots from principal component analysis based on average peak area ratios (vs. IS) of lipids listed in Supplementary Table S3, showing the statistical difference of lipid levels for (**a**) gastrocnemius and (**b**) soleus tissue samples.

From analysis of each pooled tissue sample, molecular structures of 314 lipids were identified from gastrocnemius and soleus samples, and 280 lipids were quantified using the SRM method. Quantitative data presented in a) of Supplementary Table S2 show the average peak area values [vs. internal standard (IS)] measured from each animal sample using SRM-based quantitation with nUPLC–ESI–MS/MS, as described in the experimental section. Lipid molecules exhibiting >3-fold changes in the peak area ratio with a p-value < 0.01 between C and D groups are written in bold in Supplementary Table S2a. The number of lipid species with significant changes in each lipid category is listed in Table 1 together with the total number of quantified lipids in each subclass, including the total identified lipids. Because quantification of PC and PE species was performed by detecting characteristic fragment ions ([Pcho + H]^+^ for PC and [M + H−141]^+^ for PE), few geometrical isomers that were not completely separated (i.e., 16:0/18:3-PC and 16:1/18:2-PC) were expressed with the total length of the acyl chain as 34:3-PC. Molecular structures of PC and PE species belonging to each molecule expressed with a combined chain structure (i.e., 34:3-PC) are tabulated in b) and c) of Supplementary Table S2. Therefore, numbers of quantified PC and PE molecules in Table 1 were less than those of identified species. Table 1 shows that 46 lipids from the gastrocnemius and 59 from the soleus were significantly different in diabetic rats (>3-fold changes with p < 0.01 between C and D groups). These species were selected to observe a statistical difference using principal component analysis (PCA) in Fig. 2 based on average peak area ratios (vs. IS) from all 4 groups of a) gastrocnemius and b) soleus samples from species listed in Supplementary Table S3. The score plot a) shows that C and CX were scattered without a clear isolation of each region (D and DX were similar). However, the plot b) for soleus samples indicates that D and DX were clustered at different regions and both were separated from C and CX. Thus, it can be explained that lipid patterns of both muscle tissues from diabetic rats clearly differed from those of lean controls. Moreover, PCA plots confirmed that treadmill exercise had greater effects on lipids in the soleus than in the gastrocnemius in diabetic rats. This is confirmed with the number of species that are significantly altered after exercise (listed in Table 1), but this finding will be examined in detail in subsequent data analysis. For species exhibiting significant changes (>3-fold) in their relative amounts in the D group in comparison with those in the C group, data were expressed as the peak area ratio relative to the C or D group for each lipid species for both tissue types in the D, DX, and CX groups in Supplementary Table S3. “Percentage (%) in class” in Supplementary Table S3 denotes the relative abundance of a species in each head group (e.g., PC, PE, PG) based on the peak area. For instance, the ratio of 18:3-LPC species in the gastrocnemius between D and C groups was calculated as 3.20 ± 0.34, indicating that the amount of 18:3-LPC in the D group increased by 3.20-fold compared with that in the C group; however, the relative abundance of this species was ~0.01%, which is relatively low among PC species identified in this study. High abundance in this study was defined in each head group as a relative peak area of a species in each head group of more than 1/n, where n is the number of species in each head group.

### Effect of treadmill exercise on lipid profiles

**Figure 3:**
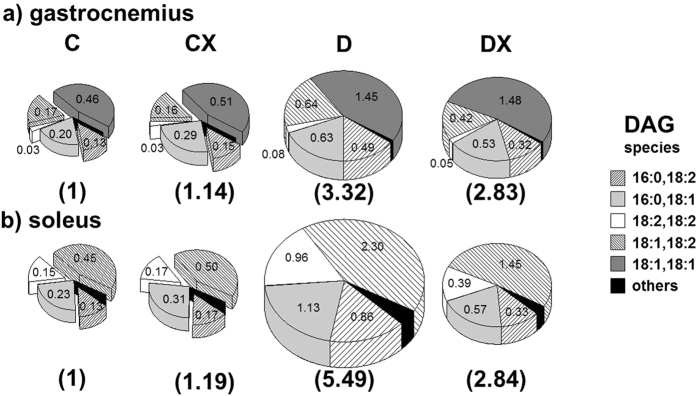
Venn diagrams presenting the changes in the relative levels of DAG species (>3-fold difference between the D and C groups with p < 0.01 according to the Mann–Whitney U test) for (**a**) gastrocnemius and (**b**) soleus samples in all rat groups (C, CX, D, and DX). The numbers in the parenthesis below each pie plot denote the total level of DAG species relative to that in group C, which was set to 1.

The number of species showing significant changes (>2 fold, p < 0.05) in diabetes after exercise (DX/D) was listed in Table 1 showing the influence of exercise on those species with significant changes (D/C > 3 fold) in D group. These species are marked as bold in DX/D column of Supplementary Table S3. Species with >2-fold changes in DX/D were found to be 31 out of 59 in soleus (31 out 59) but the numbers are less (8 out of 46) in gastrocnemius. Most of species influenced by exercise were decreased after exercise. These show the greater effect of exercise on lipids of soleus than those of gastrocnemius. While most species listed in Supplementary Table S3 were relatively low in abundance, a highly abundant species was denoted with the relative abundance value as underlined. Most highly abundant PLs displaying >3-fold changes in group D (Supplementary Table S3) did not exhibit a noticeable change in their levels after exercise (based on data of DX/C groups), excluding some Cers and DAGs. For instance, (18:2,18:2)-DAG was a highly abundant lipid displaying significant changes after exercise because its relative abundance in the soleus was calculated as 9.90% and the relative ratio of (18:2,18:2)-DAG was decreased from 6.54 ± 1.54 (D/C) to 2.62 ± 0.55 (DX/C) after exercise (p < 0.01 between D and DX groups). The patterns of changes for DAG species listed in Supplementary Table S3 were plotted using Venn diagrams in Fig. 3, with the relative amounts of DAG species in different rat groups expressed using different pie sizes. Acyl chain structures of DAGs in Fig. 3 were (16:0,18:2), (16:0,18:1), (18:2,18:2), (18:1,18:2), (18:1,18:1), and few low-abundance species. The total amount of DAGs was set to 1 for the C group, and the amount in other rat groups was expressed relative to this value (numbers in parenthesis). By comparing these numbers, the total amount of significantly changed DAG species in the diabetes group was increased by >3-fold for both tissues, but this value declined more strongly after exercise in the soleus than in the gastrocnemius. Moreover, exercise did not significantly affect the relative amount of DAG species in the control group.

**Figure 4:**
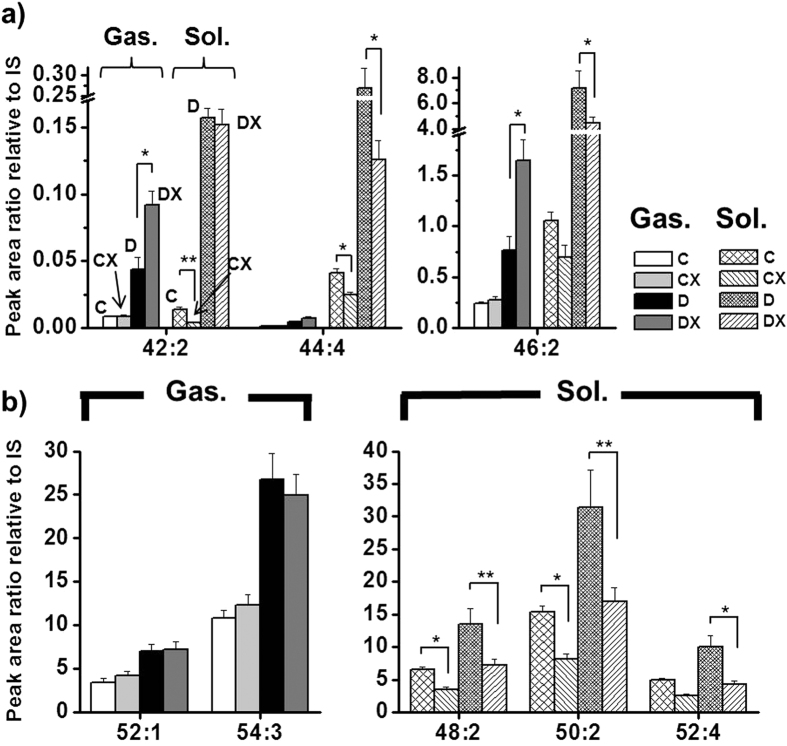
Changes in peak area ratios (relative to IS) of (**a**) relatively low-abundance but significantly altered (>3-fold with p < 0.01) TAG species and (**b**) highly abundant TAG species (>2-fold) in both tissue types between group C and D. The species marked with a single asterisk (*) showed p < 0.05 while those with a double asterisks (**) showed p < 0.01 between the D and DX groups.

Regarding TAG species, opposite trends were observed between the 2 tissue types. TAG species listed in Supplementary Table S3 were relatively low in abundance, although their levels changed by >3-fold in the diabetes group. For instance, 42:2-, 44:4-, and 46:2-TAG were relatively low-abundance species; however, their levels increased by no more than 1.5–2-fold (based on DX/D) after exercise in the gastrocnemius but decreased in the soleus (Fig. 4a). Similarly, levels of highly abundant species such as 52:1- and 54:3-TAG in the gastrocnemius and 48:2-, 50:2-, and 52:4-TAG in the soleus (Fig. 4b) were elevated by approximately 2-fold in diabetic animals; however, their levels were unchanged or elevated after exercise in the gastrocnemius but were lower in the soleus in most cases. Thus, TAGs were less influenced by treadmill exercise in the gastrocnemius than in the soleus. The possible combinations of acyl chain structures for all identified TAG species are listed in Supplementary Table S2d.

**Figure 5:**
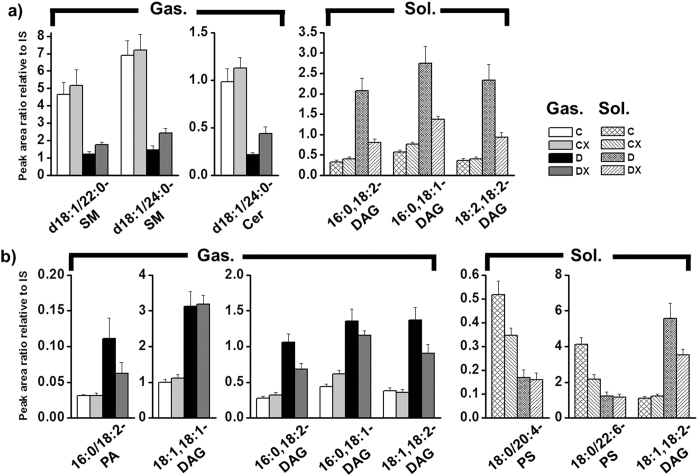
Changes in relative peak area ratios (vs. IS) of highly abundant, diabetes-stimulated lipids (C vs. D > 3-fold with p < 0.01) from Supplementary Table S3 displaying (**a**) exercise dependence and (**b**) exercise independence.

To examine diabetic lipids in depth, peak area values of some highly abundant species that were stimulated by diabetes in Supplementary Table S3 were plotted as exercise-dependent diabetic lipids in plot a) and exercise-independent diabetic species in plot b) in Fig 5. Levels of the 2 SM species (d18:1/22:0 and d18:1/24:0) and d18:1/24:0-Cer were significantly reduced in the gastrocnemius in the diabetes group. Although the degree of recovery was relatively weak in comparison with that in the control group, these changes were found to be statistically meaningful. Three DAGs [(16:0,18:2), (16:0,18:1), and (18:2,18:2)] from soleus samples displayed nearly full recovery after exercise. However, 16:0/18:2-PA and 4 DAGs [(18:1,18:1), (16:0,18:2), (16:0,18;1), and (18:1,8:2)] from gastrocnemius samples and 2 PSs (18:0/20:4 and 18:0/22:6) and (18:1,18:2)-DAG from soleus samples exhibited no change or slight decreases after exercise, but the extent of the decrease was not statistically meaningful (p < 0.05 between D and DX groups); thus, they were considered exercise independent. The specified lipid molecules in Fig. 5 were relatively high-abundant species, and the entire list of lipids displaying recovery or exercise-independent trends is sorted in Table 2. Briefly, 10 and 39 lipids from gastrocnemius and soleus samples, respectively, responded to exercise, whereas 33 and 19 species, respectively, were exercise independent. It was noted that 2 highly abundant DAGs (16:0,18:2 and 16:0,18:1) in both tissue samples displayed exercise dependence in the soleus, but they were found to be exercise independent in the gastrocnemius. Meanwhile, (18:1,18:2)-DAG was exercise independent in both tissues.

## Discussion

The present experiments using diabetic rats revealed that chronic exercise induces a decrease in most lipid levels. PS functions as a signaling molecule for macrophages during cell apoptosis by translocating to the outer leaflet of the cellular membrane, whereas this moiety typically remains in the inner leaflet. In our study, levels of 4 PS species (16:0/22:6, 18:0/20:4, 18:1/22:6, and 18:0/22:6) were significantly lower (>3-fold) in the soleus of diabetic rats (Supplementary Table S3), which can be explained as an abnormality in cell apoptosis in diabetic animals. By comparing the ratio of muscle tissue weight to body weight between the 2 muscle types listed in Supplementary Table S1, it is obvious that the relative weight of the soleus increased significantly in the DX group in comparison to the D group, but the relative weight of gastrocnemius increased slightly in DX group in comparison to the D group. However, their change was not significant as their p-value was larger than 0.05. Also, no change (p > 0.05) was observed after exercise in either muscle type in control animals. This corresponds to lipid patterns observed following exercise in Fig. 2, indicating that the data points of the DX group approach those of the CX group, whereas those of groups C and CX are not distinguishable from each other for both muscle types. These findings support that changes in lipid profiles induced by diabetes are influenced to some extent by treadmill exercise in the soleus. Also, the effect of exercise on C and D was very similar to one another as most of lipids showed the same trends in both groups after exercise (CX and DX). The magnitudes of increase/decrease was significantly larger in DX but CX mostly showed the same trends by decreasing or increasing slightly after exercise.

We found that levels of most DAG, TAG, and LPL species in skeletal muscle are increased in the presence of diabetes. DAG interrupts insulin signaling, and its levels are increased by insulin resistance^33^,^34^. As not all DAG and TAG species are directly associated with insulin resistance, it is meaningful to quantify individual lipid species related to diabetes according to the influence of treadmill exercise on their recovery. While the levels of 10 DAGs from the gastrocnemius and 13 from the soleus were increased by >3-fold with diabetes, it was noted that eight DAG species among them were commonly found in both tissue types; in particular, (16:0,18:2), (16:0, 18:1), and (18:1,18:2) relatively high in abundance among them. Increases in DAG levels are known to activate PKC, which in turn activates phospholipase A2 (PLA2)^35^,^36^. Because PLA2 cleaves the sn-2 acyl chain from PLs, elevated DAG levels may result in an increase in LPL levels, in line with our results.

While the gastrocnemius consists of red (type I) and white (type II) muscle fibers, which are known as slow and fast oxidative muscle fibers, respectively, the soleus is mainly composed of red muscle that responds to aerobic exercise. Therefore, aerobic exercise on a treadmill results in significant changes in soleus lipid levels. The data in Table 2 illustrate that soleus lipid levels are more strongly influenced by exercise than those in the gastrocnemius because levels of 10 lipids in the gastrocnemius and 39 lipids in the soleus tended to recover toward control levels, whereas 33 lipids in the gastrocnemius and 19 lipids in the soleus were not influenced by exercise. In particular, levels of most DAGs, TAGs (except 42:2-, 42:3-, 42:1-, and 42:0-TAGs), and Cer in the soleus (Table S3) tended to recover toward control levels, but similar trends were not observed in the gastrocnemius. For instance, some highly abundant DAGs [(16:0,18:2) and (16:0,18:1)] exhibited an opposite trend in the gastrocnemius in that their levels recovered toward control levels. This is illustrated by plotting the fold changes (CX/C and DX/D) of species marked as bold (in Supplementary Table S3) along with the species showing recovery trends as described above marked with asterisk (*) in Supplementary Fig. S4. Cer levels are known to increase when insulin resistance occurs. In this study, d18:1/16:0-Cer and d18:1/18:1-Cer levels in the soleus were increased in the D group, whereas d18:1/24:1-Cer and d18:1/24:0-Cer levels were decreased in the gastrocnemius, supporting that insulin resistance may influence lipid levels differently between the muscle types. The changes in Cer levels upon exercise are unclear, as it was reported that the total Cer level did not change after chronic exercise in the rat gastrocnemius^37^. However, another study indicated that acute prolonged exercise improved the Cer level of human skeletal muscle^38^. Despite differences in types of Cer species between humans and rats, the discrepancy of the 2 results may have been influenced by the duration and intensity of exercise.

The present study demonstrated that lipid levels in diabetic rat muscle appear to recover to control levels after aerobic exercise, with a larger degree of change observed in the soleus than in the gastrocnemius, supporting that chronic exercise has beneficial effects on the soleus in diabetic animals. Because this study utilized a chronic 6-week training protocol, further experiments are required to evaluate the effect of different exercise protocols (type, intensity, frequency, and duration) on diabetes together with different dietary states.

## Methods

### Materials and Reagents

To determine the nLC–ESI–MS/MS run condition, 28 lipid standards from Avanti Polar Lipids, Inc. (Alabaster, AL, USA) were utilized: 12:0-LPC, 16:0-LPC, 18:1-LPC, 12:0/12:0-PC, 13:0/13:0-PC, 16:0/16:0-PC, 18:1/18:0-PC, 20:0/20:0-PC, 14:0-LPE, 18:0-LPE, 12:0/12:0-PE, 14:0/14:0-PE, 18:0/22:6-PE, 12:0-LPG, 15:0/15:0-PG, 18:0-LPS, 18:0/18:0- PS, 16:0/18:2-PI, 18:0-LPA, 12:0/12:0-PA, (18:1)_4_-CL, d18:0/12:0-SM, d18:1/24:0-SM, d18:1/12:0-MHC, d18:1/16:0-DHC, d18:1/12:0-Cer, 16:0/18:1-DAG, and 16:0/16:0/18:1-TAG. All solvents used in this study were of HPLC grade. H_2_O, CH_3_CN, CH_3_OH, isopropanol, and methyl-tert-butyl ether were obtained from Avantor Performance Materials (Center Valley, PA, USA), and CHCl_3_ was purchased from Sigma-Aldrich (St. Louis, MO, USA). NH_4_HCO_2_ and NH_4_OH (Sigma-Aldrich) were used as ionization modifiers and added to LC mobile phase solutions. All PL standards were diluted with a binary solvent mixture (9:1, v/v, CH_3_OH:H_2_O). Silica capillary tubes used for capillary columns, and all connections were purchased from Polymicro Technology, LLC (Phoenix, AZ, USA), with all tubes having inner diameters of 20, 50, 75, or 100 μm (outer diameter of 360 μm for all).

### Animals and training plan

Male ZDF (fa/fa) rats and control ZLC (fa/+) rats (Genetic Models Inc., Indianapolis, IN, USA) were housed in a conventional state maintained at 24 ± 2 °C with a 12-h/12-h light dark cycle and fed a standard chow diet (Purina Korea, Korea) and tap water. All experimental methods were carried out in accordance with the approved guidelines of “Guide for Animal Experiments” (Edited by Korean Academy of Medical Sciences) and all experimental protocols were approved by the Institutional Animal Care and Use Committee (IACUC) of Seoul National University (Seoul, Korea, Permit number : SNU-131121-1).

Rats were separated into 4 groups for lipid analysis: C (n = 14), CX (n = 15), D (n = 9), and DX (n = 12). At 8 weeks of age, CX and DX rats were placed on a motorized rodent treadmill (1 animal/lane). The training intensity was modified from that in a previous study^39^ into a reduced scale as follows: The treadmill exercise began with an intensity of 10 m/min (a prescribed speed for 5 min and warm-up/cool-down for 5 min) for 10 min/day 5 times a week for the first week, following which the intensity was gradually increased to 14.5 m/min for 50 min/day (a prescribed speed for 45 min and warm-up/cool-down for 5 min) 5 times a week for the next 6 weeks (see Supplementary Table S4 for the detailed schedule). Over this duration, the speed was gradually increased from 5 to 10 m/min at 0% grade, as tolerated by rats. Animals in the sedentary groups were also handled and put on the treadmill for 5–10 min/session during this intervening period. Body weights were measured during exercise periods every week. All animals were sacrificed after 14 weeks. Blood (fast glucose) was sampled via heart puncture before sampling tissue using a 21 G needle and analyzed using an Accu-Chek glucose analyzer (Roche Diagnostics Ltd., Mannheim, Germany). Animals were sequentially perfused transcardially with 0.9% NaCl solution, and white adipose tissue from epididymal fat, liver, gastrocnemius muscle, and soleus muscle were removed. Tissue samples were weighed immediately, frozen with liquid nitrogen, and stored at −80 °C for further investigation. Weighed values of tissues and organs are listed in Supplementary Table S1.

### Lipid extraction from tissue samples

For the global search of lipid molecular species from tissue samples, a small portion of dried tissue samples from individual animals was combined to prepare a 15-mg pooled tissue sample representing each group (gastrocnemius and soleus samples from the C, CX, D, and DX groups). For quantitative analysis of each animal sample (a total of 100 samples), 15 mg was separately extracted from each tissue sample. Following this, 15 mg of a dried tissue sample was rehydrated with 150 μL of 0.1 M PBS solution and then sonicated with a tip for 5 min. Next, lipid extraction was performed by following the modified Folch method using MTBE/CH_3_OH^40^. The sonicated mixture was mixed with 300 μL of CH_3_OH followed by vortexing for 10 min, and 1000 μL of MTBE was added to the mixture. The mixture was vortexed for 1 h, and 250 μL of H_2_O was added, followed by centrifugation at 1000 × *g* for 10 min. The upper organic layer was taken to a new tube. The remaining layer was mixed with 300 μL of CH_3_OH and sonicated for 2 min. After centrifuging at 1000 × *g* for 10 min, the upper layer was pipetted to the previous organic extract. The combined extract was dried under vacuum centrifuge overnight, weighed, and the final dried powder was dispersed with CHCl_3_:CH_3_OH (3:7, v/v) for storage at −30 °C. For nLC–ESI–MS/MS analysis, each mixture was diluted to a concentration of 5 μg/μL with CH_3_OH:H_2_O (9:1, v/v). As each individual contains different amount of lipids within the same amount of tissue, the extracted lipids were diluted to the same concentration rather than dissolving them in the same volume of dispersing solvent, to prevent the low-abundant lipids from being not detected by MS. In order to adjust the total lipid concentration, the dilution factor, volume of final solution added after the extraction, of each sample was utilized to calculate the final amount of lipids by multiplying the peak areas by the dilution factor.

### nLC–ESI–MS/MS

For the global search of lipid molecules from each sample group (8 groups in total), a model 1200 capillary pump system equipped with an autosampler (Agilent Technologies, Palo Alto, CA, USA) coupled to an LTQ Velos ion trap mass spectrometer (Thermo Scientific, San Jose, CA, USA) was utilized with an analytical column. For targeted quantitation of identified lipids from global search, the SRM-based quantitation method was employed with UPLC–ESI–MS/MS using a nanoACQUITY UPLC system (Waters, Milford, MA, USA) equipped with an autosampler and a TSQ Vantage triple-stage quadrupole MS system (Thermo Scientific). The list of product ions used for SRM along with each collision energy value are listed in Supplementary Table S5. Details are found in Supplementary Information online.

## Additional Information

**How to cite this article**: Lee, J. C. *et al*. Evaluation of treadmill exercise effect on muscular lipid profiles of diabetic fatty rats by nanoflow liquid chromatography–tandem mass spectrometry. *Sci. Rep*. **6**, 29617; doi: 10.1038/srep29617 (2016).

## Supplementary Material

Supplementary Information

## Figures and Tables

**Figure 1:**
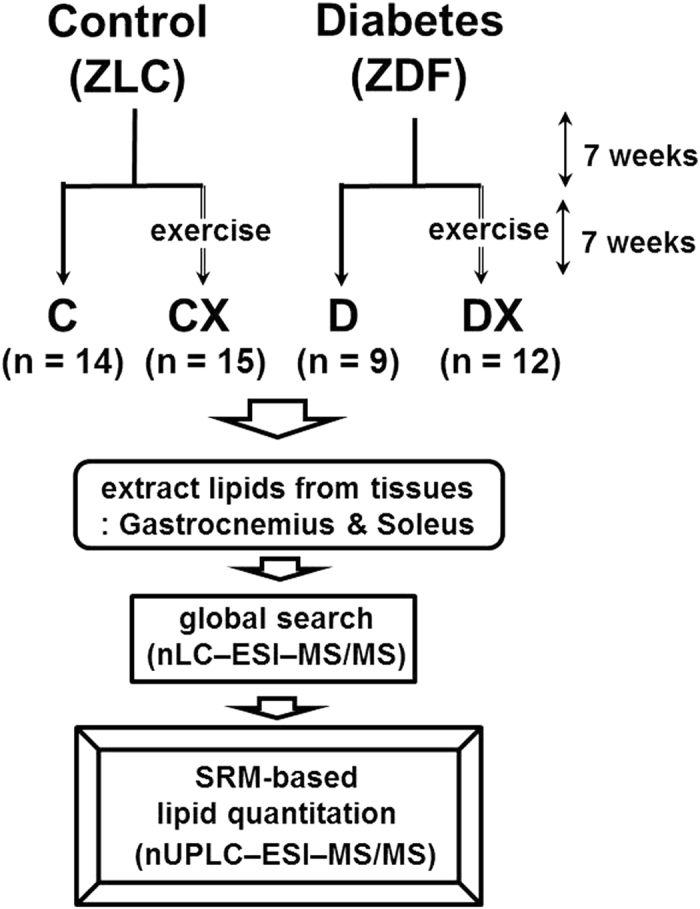
Schemes of experiments for the different rat groups depending on exercise periods and quantitative analysis.

**Table 1:** Number of lipids identified via global searching by nLC–ESI–MS/MS in each lipid class, number of lipids quantified by nUPLC–ESI–MS/MS using the SRM method, number of lipids displaying >3-fold differences between the Control (C) and Diabetes (D) groups for each tissue type (gas.: gastrocnemius, sol.: soleus), number of species showing >2-fold change after exercise among those of D/C > 3 fold.

	Identified	Quantified by SRM	>3-fold change in D/C		>2-fold change in DX/D among D/C > 3 fold	
			Gas.	Sol.	Gas.	Sol.
LPC	12	12	4	9	0	6
PC	57	34	4	4	3	3
LPE	11	11	2	3	0	2
PE	34	23	1	1	1	1
LPG	6	6	2	1	0	1
PG	15	15	1	4	0	0
LPI	7	7	2	1	0	0
PI	22	22	1	0	0	0
LPS	2	2	1	1	0	0
PS	23	23	0	4	0	0
LPA	3	3	1	0	0	0
PA	2	2	1	0	0	0
SM	9	9	5	0	0	0
Cer	8	8	2	2	1	2
MHC	2	2	0	0	0	0
DAG	37	37	10	13	0	12
TAG	64	64	9	16	3	4
Total	314	280	46	59	8	31

**Table 2:** List of diabetes-stimulated lipids according to their a) dependence and b) independence on exercise. Species written in bold were relatively high in abundance among their corresponding head group species.

Gastrocnemius		Common from both	Soleus	
a)				
38:2-, 40:8-, 36:0-	PC		30:0-, 42:1-	PC
34:0-	PE	34:0-PC	20:3-, 16:0-, 18:0-, 20:4-, 22:6-, 22:5-, 22:4-	LPC
**d18:1/22:0-**, d18:1/23:0-, d18:1/24:2-, ** d18:1/24:0-**	SM		20:5-LPE, 22:5-LPE, 16:1-LPG	
**d18:1/24:0-**	**Cer**		d18:1/16:0-, d18:1/18:1**-**	Cer
			(14:1,16:0), (16:1,18:3), (16:0,18:3),**(16:0,18:2), (16:0,18:1)**, (18:2,18:3),**(18:2,18:2)**, (14:0,16:0), (18:0,18:0),(18:0,20:4), (18:1,22:4), (18:0,22:4)	DAG
			44:4-, 46:2-, 54:7-, 56:6-, 42:4-, 46:5-,46:4-, 46:1-, 46:0-, 48:3-, 48:0-, 50:4-	TAG
b)				
18:3-, 20:3-	LPC		16:0/16:0-, 20:4/16:0-, 18:2/18:0-	PG
16:1-, 20:5-	LPE	16:1-LPC,	16:0/22:6-, **18:0/20:4-**, 18:1/22:6-,**18:0/22:6-**	PS
14:0-, 16:1-	LPG	18:2-LPC	36:0-PC, 22:4-LPE	
18:2-LPI, 18:2-LPA. 18:2/20:4-PI, ** 16:0/18:2-PA**, d18:1/20:0-SM, d18:1/24:1-Cer		20:3-LPI, 18:1-LPS, 22:6/22:6-PG		
**(18:1,18:1)**, (16:1, 22:6), (14:1,16:0), (16:1,18:3), (16:0,18:3), **(16:0,18:2)**, ** (16:0,18:1)**, (18:2,18:3), (18:2,18:2)	DAG	**(18:1,18:2)-DAG**		
44:3-, 56:7-, 50:0-, 44:4-, 54:7-, 56:6-	TAG		42:2-, 42:3-, 42:1-, 42:0-	TAG
