# Supplementary material for: Evaluation of treadmill exercise effect on muscular lipid profiles of diabetic fatty rats by nanoflow liquid chromatography–tandem mass spectrometry
Source: Sci Rep. 2016 Jul 8;6:29617. doi: 10.1038/srep29617 (PMC4937420; doi:10.1038/srep29617)
Supplement: Supplementary Information [file srep29617-s1.pdf]

Supplementary Information

Evaluation of treadmill exercise effect on muscular lipid profiles of diabetic fatty rats by nanoflow liquid chromatography–tandem mass spectrometry

Jong Cheol Lee,[1] Il Yong Kim,[2,3] Yeri Son,[2,3] Seul Kee Byeon,[1] Dong Hyun Yoon,[3,4] Jun Seok Son,[3,4] Han Sol Song,[3,4] Wook Song,[3,4,5] Je Kyung Seong,[2,3*] and Myeong Hee Moon[1,*]

[1]Department of Chemistry, Yonsei University, Seoul, 03722, Korea

[2]College of Veterinary Medicine, Seoul National University, Seoul, 08826, Korea
[3]Korea Mouse Phenotyping Center (KMPC), Seoul National University, Seoul, 08826, Korea
[4]Health and Exercise Science Laboratory, Institute of Sport Science, Seoul National University College of Education, Seoul, 08826, Korea
[5]Institute on Aging, Seoul National University College of Medicine, Seoul, 08826, Korea.

Table of Contents

nLC–ESI–MS/MS of lipids 2
Structural determination of DAG and TAG 4
Figure S1 5
Figure S2 6
Figure S3 7
Figure S4 8
Table S1 9
Table S2 10
Table S3 25
Table S4 29
Table S5 29

nLC–ESI–MS/MS

For the global search of lipids by nLC-ESI-MS/MS (ion trap), the column was prepared in the laboratory by packing Watcher ODS-P (3 μm and 100 Å) C18 resins (Isu Industry Corp. Seoul, Korea) into a pulled tip silica capillary tube (75 μm I.D. with 360 μm O.D.) under N_2 (1000 psi) to a column length of 7 cm. The pulled tip column was prepared by pulling one end of the silica tube with a flame to make a sharp needle so that ESI can be achieved without a separate emitter. The analytical column was connected to a PEEK microcross (IDEX, Oak Harbor, WA, USA), the other three ports of which were connected to a capillary tube (50 µm I.D.) from the LC pump, a Pt wire to supply electrical voltage for ESI, and a pressure capillary (20 µm I.D.) for venting. The latter was used to provide a pressure for the controlling the column flow rate at 300 nL/min by varying its length and for venting the split flow. The end of the pressure tube was attached to the on/off switching valve so that it was closed during sample loading to column and opened during LC separation. Separation of lipids by nLC was achieved with gradient elution using two mobile phases: H_2O:CH_3CN (9:1, v/v) for mobile phase A and CH_3OH:CH_3CN:isopropanol (2:2:6, v/v/v) for phase B. To both mobile phase solutions, 5 mM NH_4HCO_2 and 0.05% NH_4OH were added as modifiers, which were utilized in the positive and negative ion modes of MS analysis. The sample was loaded to an analytical column using mobile phase A with the switching valve off at 600 nL/min for 15 min. After loading, gradient elution was initiated by ramping mobile phase B from 0% to 55% for 1 min, 80% for 20 min, and 100% for 20 min. For column washing, mobile phase B was maintained at 100% for 30 min and then lowered to 0%. To minimize the dwell time during gradient elution, a flow rate of 15 µL/min was applied with the switching valve on so that a final concentration of 300 nL/min was delivered to an analytical column and the rest of the flow exited through the vent tube. The injected amount of each sample was adjusted to 10 µg based on the weights of dried lipids. Two internal standards (IS), 1 pmol of 13:0/13:0-PC for the positive ion mode and 1 pmol of 15:0/15:0-PG for the negative ion mode, were injected together with 10 µg of sample to compensate the fluctuation in MS intensity. For MS analysis, 3.0 kV of ESI voltage was applied. The mass ranges of detection were 400–1100 and 350–1100 amu for the negative and positive ion modes, respectively. For each precursor run, 3 data-dependent CID analyses were accomplished with 40% normalized collision energy. Structural determination of lipid molecules from fragment ion spectra was performed

using the LiPilot computer algorithm (1), which was developed in our laboratory and confirmed by manual examinations.

For targeted quantitation of identified lipids from the non-targeted analysis, the selected reaction monitoring (SRM)-based quantitation method was performed by UPLC–ESI–MS/MS using a nanoACQUITY UPLC system (Waters, Milford, MA, USA) equipped with an autosampler and a TSQ Vantage triple stage quadrupole MS system (Thermo Scientific). The analytical column for UPLC was prepared in our laboratory in the same manner as that described at the above, but it was packed in a pulled tip capillary (100 μm I.D.) using 1.7-μm ethylene bridged hybrid (BEH) particles (130 Å) that were unpacked from an XBridge® BEH C18 column (2.1 mm × 100 mm, Waters). Before packing, the 5-mm length of the needle tip was filled with Watchers ODS-P (3 μm and 100 Å) to make a self-assembled frit, and the rest was packed with 1.7-μm BEH particles. Configuration of the column connection with a vent tube and Pt wire, the injected amount of the lipid extract, and the type of mobile phase solutions was as described previously. Sample loading was performed at 1 μL/min of mobile phase A for 10 min, and gradient elution was initiated by increasing mobile phase B to 70% for 0.1 min, 90% for 4.9 min, and 100% for 8 min and maintained for 7 min. During gradient elution, the pump flow rate was maintained at 21 μL/min to reduce the dwell time, and the final column flow rate was adjusted to 300 nL/min. The same amount of samples (10 μg) and ISs (1 pmol) were injected for each analysis. The ESI voltage was fixed at 3.0 kV, but the collision energies for SRM analysis were varied according to lipid types as 20 (LPE and PE), 25 (DAG and TAG), 30 (Cer and MHC), 35 (LPG, PG, LPI, PI, LPS, PS, LPA, and PA), or 40 V (LPC, PC, and SM) with a scan width of *m/z* 1.0 and a scan time of 0.01 s. Targeted quantitation was performed in the positive and negative ion modes, which were repeatedly switched during a single run. Lipid species detected during a positive ion mode cycle were LPC, PC, LPE, PE, SM, Cer, and MHC for $[M+H]^+$ and DAG and TAG for $[M+NH_4]^+$, and the remaining species were detected during a negative cycle for $[M-H]^-$. The types of characteristic fragment ions for SRM were protonated phosphocholine ions (m/z 184) for LPC, PC, and SM, neutral loss of phosphoethanolamine ($[M+H-141]^+$) for LPE and PE, LCB fragments (m/z 264) for Cer and MHC, $[M+NH_4-RCOONH_4]^+$ for DAG and TAG, and carboxylate anions of acyl chains ($[R_1COO]^-$ and $[R_2COO]^-$) for the remaining lipid species detected using the negative ion mode. Statistical analyses of data were accomplished using Minitab 15 software (http://www.minitab.co.kr) for principal component analysis and SPSS software (version 20.0, IBM Corp. Armonk, NY, USA) for the Mann–Whitney U test.

Structural determination of DAG and TAG

The CID spectra in Fig. S3a show four acylium ions, $[RCO]^+$ at m/z 237.3, 239.3, 263.4, and 265.5, after the dissociation of acyl chains from TAG, corresponding to acyl chains of 16:1, 16:0, 18:2, and 18:1, respectively. Fragment ions at m/z 603.5, 601.6, 577.5, and 575.6 represent the loss of acyl chains (from 16:1 to 18:1) in the form of ammonium carboxylate as $[M+NH_4-RCOONH_4]^+$ from the ammonium adduct form of TAG. Similarly, ions clustered between m/z 313.5 and 341.5 represent the loss of two adjacent acyl chains in the form of carboxylic acid and ketene, namely $[M+NH_4-RCOONH_4-R'CH=C=O]^+$. From these spectra, the molecular structure of spectrum a) can be assigned as two isomeric TAG molecules, (16:0,18:1,18:2)-TAG and (16:1,18:1,18:1)-TAG, without a positional order of acyl chains. A slightly different fragmentation pattern was observed with DAG molecules in Fig. S3b, revealing the loss of ammonia from the ammonium adduct ion ($[M+NH_4-NH_3]^+$) at m/z 621.6, together with the loss of water ($[M+NH_4-NH_3-H_2O]^+$) at m/z 603.5 and the characteristic loss of acyl chains in the form of ammonium carboxylate as $[M+NH_4-RCOONH_4]^+$ at m/z 337.4, 339.3, and 341.3, which were obtained from the dissociation of acyl chains 18:0, 18:1, and 18:2. The CID spectra in Fig. S3b can be assigned as (18:0,18:2)-DAG and (18:1,18:1)-DAG without positional information for the acyl chains. The molecular structures of typical PLs can be readily obtained via CID experiments during nLC–ESI–MS/MS, as reported in earlier studies (refs. 23-26 in the text).

References

1. Lim, S., Byeon, S. K., Lee, J. Y. & Moon, M. H. Computational approach to structural identification of phospholipids using raw mass spectra from nanoflow liquid chromatography-electrospray ionization-tandem mass spectrometry. *J. Mass Spectrom.* **47,** 1004-1014, (2012).

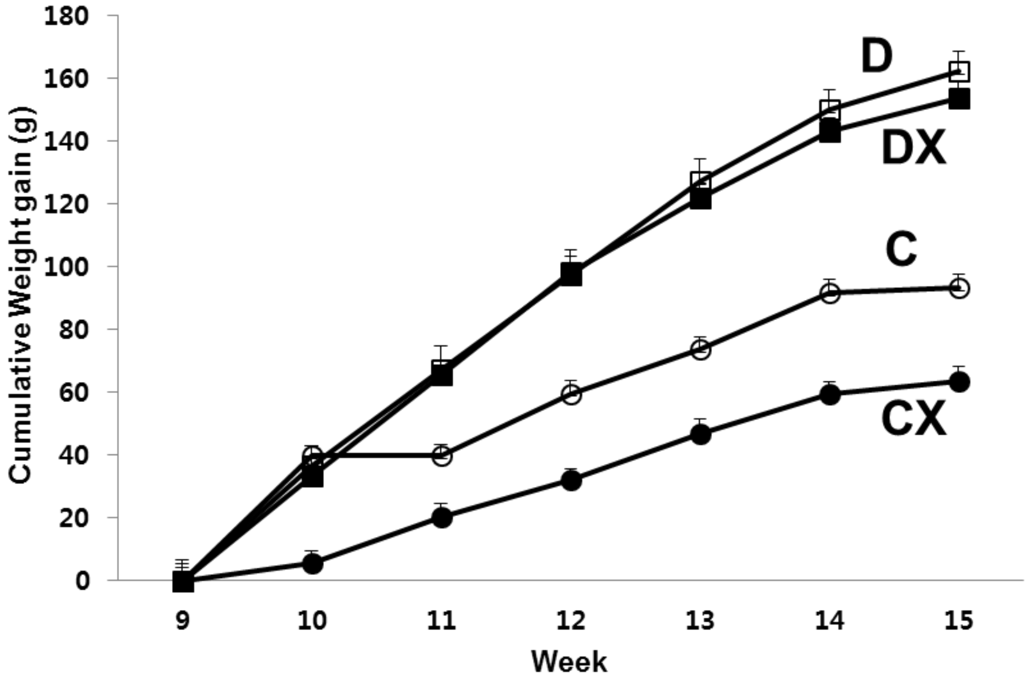

	ZLC		ZDF	
	C	CX	D	DX
Weight at 9th week (g)	178.67 ± 4.22	184.80 ± 6.50	246.40 ± 5.34[a]	235.00 ± 2.05[b]
Weight at 15th week (g)	272.00 ± 4.41	248.40 ± 4.71	408.80 ± 6.25[a]	388.83 ± 4.79[bc]
difference (g)	93.33 ± 4.31	63.60 ± 5.42[a]	162.40 ± 4.75[a]	153.83 ± 5.83[b]

[a]$P < 0.05$ vs. C group; [b]$P < 0.05$ vs. CX group; [c]$P < 0.05$ vs. D group.

Figure S1. Plot showing the cumulative body weight gains of animals during the period of exercise and measured weight values at weeks 9 and 15

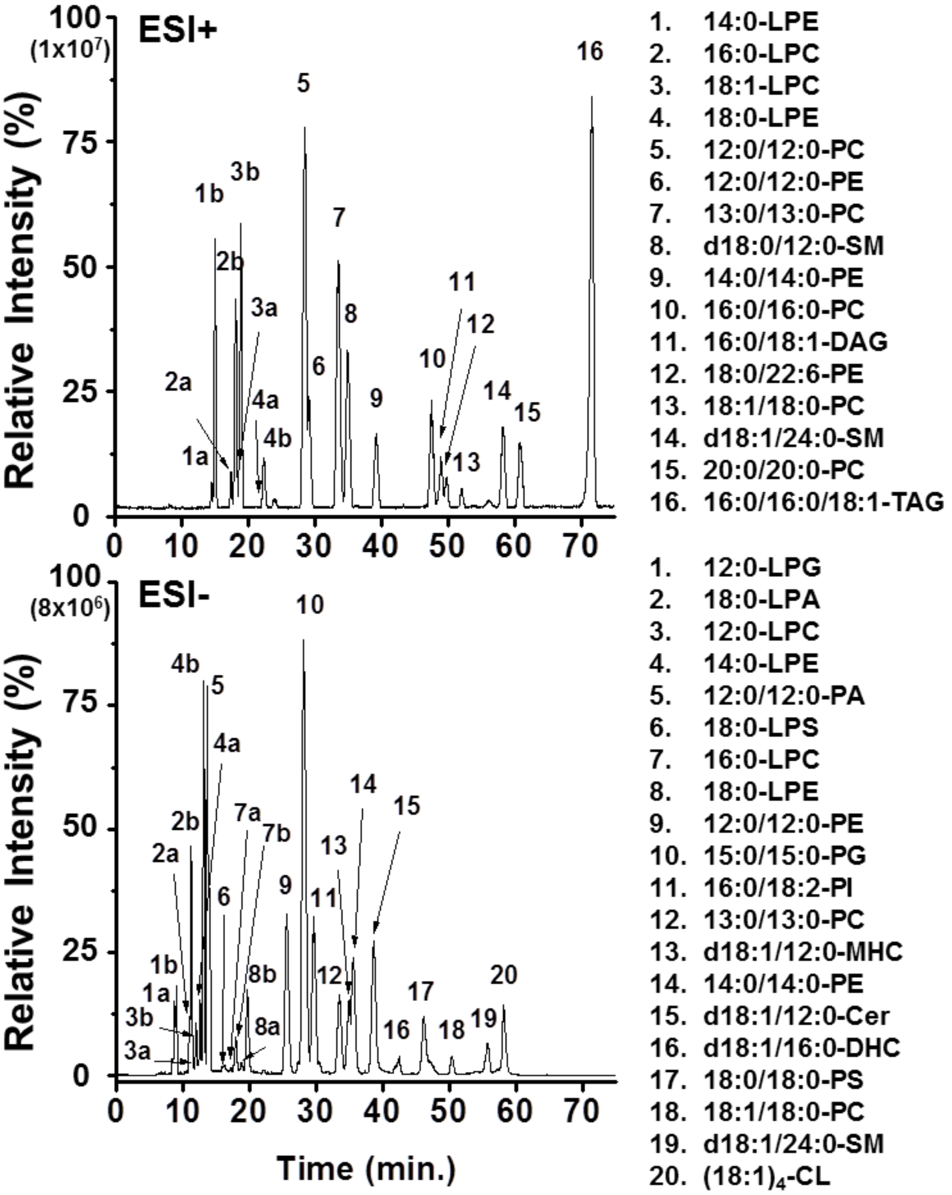

Figure S2. Base peak chromatograms of standard lipid mixtures obtained by nLC–ESI–MS/MS in the positive (top) and negative (bottom) ion modes.

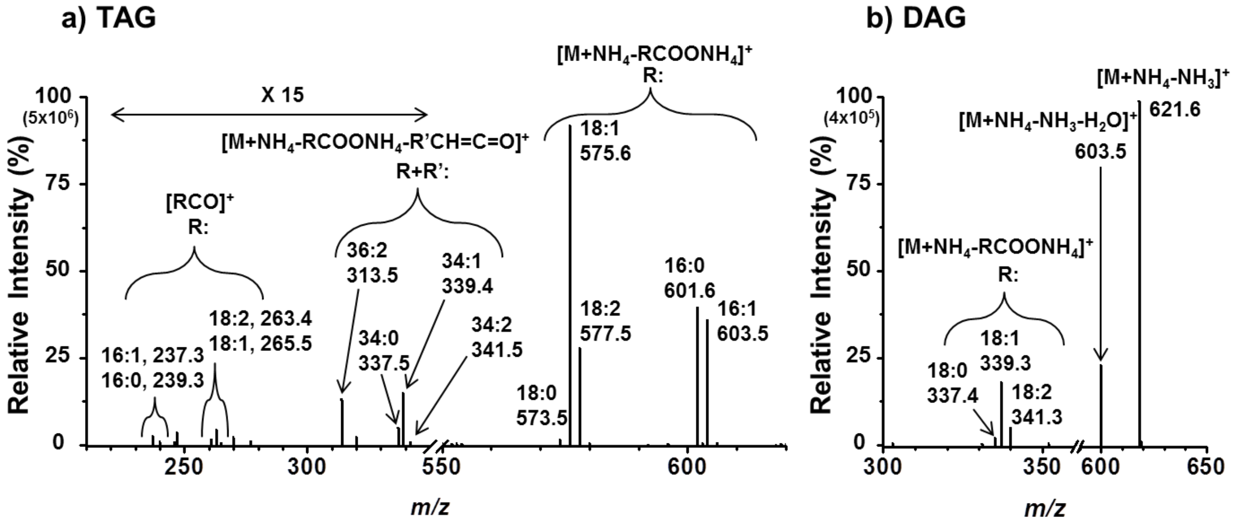

Figure S3. CID spectra assigned for a) 52:3-TAG isomeric forms (m/z 874.7, $[M+NH_4]^+$, t_r = 60.86 min) and b) 36:2-DAG (m/z 638.5, $[M+NH_4]^+$, t_r = 49.57 min) obtained by nLC–ESI–MS/MS analysis of a pooled soleus lipid sample from group D.

176

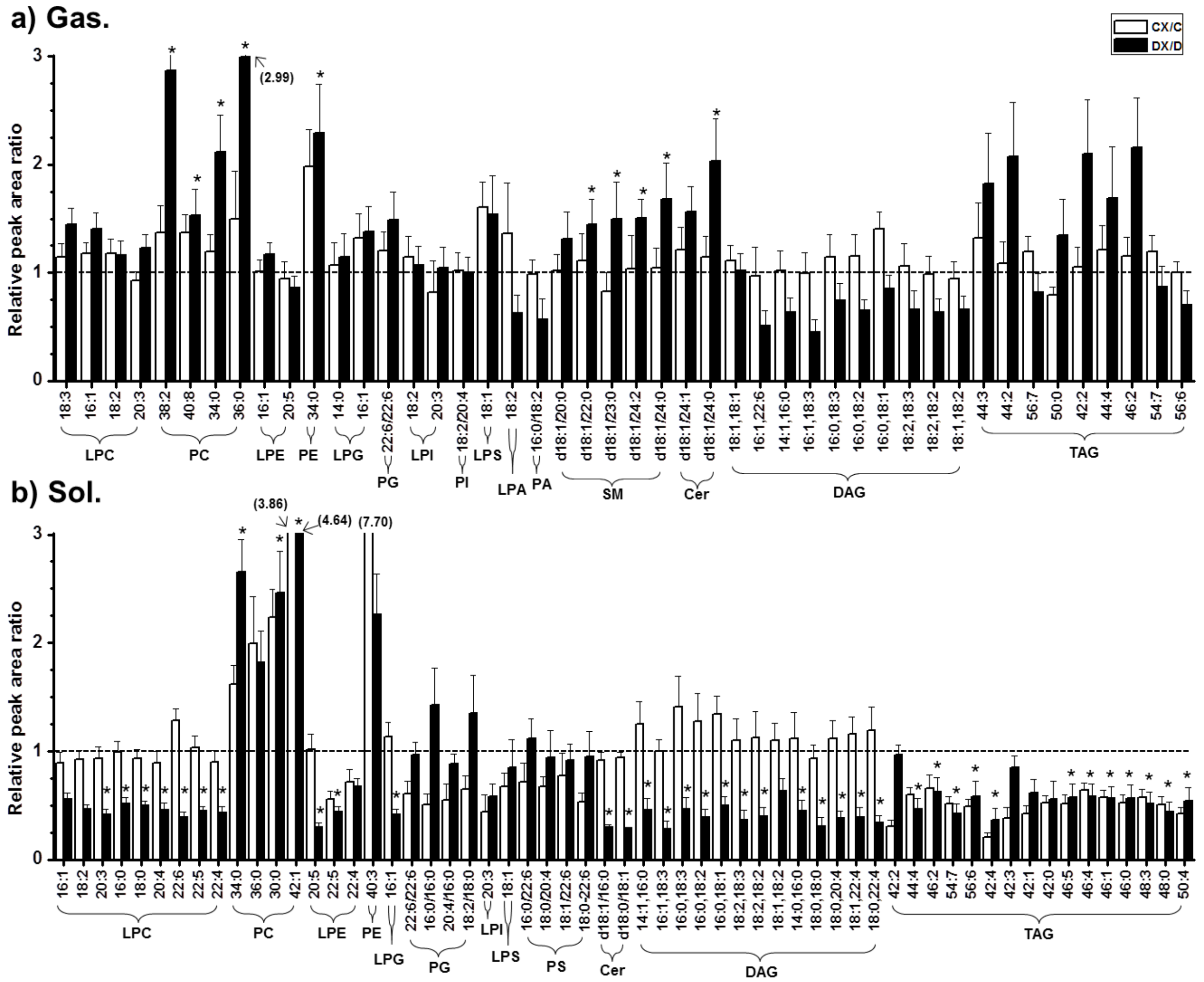

Figure S4. Fold changes (CX/C and DX/D) of lipids species (D/C>3-fold with p<0.01) from Table S3. The species marked with asterisk (*) showed recovery trends sorted in Table 2a.

Table S1. Fasted body and tissue weights and fasted plasma glucose levels after 7 weeks of physical exercise (percentage (%) values are based on the relative amount of each organ or tissue relative to body weight).

	ZLC		ZDF	
	C	CX	D	DX
body weight (g)	264.25 ± 4.00	244.28 ± 3.53[a]	396.52 ± 6.35[a]	387.25 ± 5.82[b]
epididymal fat (g)	1.73 ± 0.06	1.54 ± 0.07	8.75 ± 0.40[a]	8.05 ± 0.48[b]
(%)	0.66 ± 0.02	0.63 ± 0.03	2.20 ± 0.07[a]	2.07 ± 0.10[b]
liver (g)	7.27 ± 0.14	6.72 ± 0.11[a]	16.89 ± 1.01[a]	15.83 ± 0.67[b]
(%)	2.75 ± 0.08	2.75 ± 0.04	4.26 ± 0.24[a]	4.08 ± 0.12[b]
gastrocnemius (g)	2.57 ± 0.04	2.36 ± 0.01[a]	2.19 ± 0.04	2.35 ± 0.07[bc]
(%)	0.97 ± 0.01	0.97 ± 0.01	0.55 ± 0.01[a]	0.61 ± 0.03[b]
soleus (g)	0.26 ± 0.01	0.23 ± 0.00[a]	0.25 ± 0.01[a]	0.27 ± 0.00
(%)	0.10 ± 0.00	0.10 ± 0.00	0.06 ± 0.00[a]	0.07 ± 0.00[bc]
fasted blood glucose (mg/dL)	165.67 ± 8.98	144.40 ± 0.93	226.40 ± 7.78[a]	210.00 ± 12.15[c]

[a]$p < 0.05$ vs. C group; [b]$p < 0.05$ vs. CX group; [c]$p < 0.05$ vs. D group

Table S2. a) Peak area of lipid species (vs. IS) from both tissue samples of the control (C), control after exercise (CX), diabetes (D), and diabetes after exercise (DX) groups obtained by nLC–ESI–MS/MS (The molecular structures of PC, PE, and TAG species are expressed with the total chain lengths, but detailed molecular structures of corresponding isomers for PC and PE identified from qualitative analysis are listed in b) and c) and the possible combinations of acyl chain structures for TAG species are listed in d). Species marked with [a, b, c] were isomers quantified individually because these isomers were separated as different molecular structures in nUPLC–ESI–MS/MS.).

a)

class	acyl chain	*m/z*	gastrocnemius				soleus			
			C	CX	D	DX	C	CX	D	DX
**LPC**	14:0	468.3	0.02 ± 0.00	0.03 ± 0.00	0.05 ± 0.00	0.05 ± 0.00	0.07 ± 0.00	0.06 ± 0.00	0.18 ± 0.02	0.08 ± 0.01
	**16:1**	**494.3**	**0.08 ± 0.00**	0.09 ± 0.01	**0.47 ± 0.04**	0.66 ± 0.05	**0.23 ± 0.02**	0.21 ± 0.02	**1.29 ± 0.12**	0.72 ± 0.04
	16:0	496.4	3.77 ± 0.22	4.55 ± 0.23	8.97 ± 0.77	7.89 ± 0.61	**7.42 ± 0.56**	7.33 ± 0.54	**35.58 ± 2.95**	18.34 ± 1.24
	**18:3**	**518.5**	**0.02 ± 0.00**	0.02 ± 0.00	**0.07 ± 0.01**	0.10 ± 0.01	0.12 ± 0.01	0.09 ± 0.01	0.33 ± 0.03	0.12 ± 0.01
	**18:2**	**520.5**	**2.10 ± 0.17**	2.48 ± 0.19	**9.83 ± 0.95**	11.37 ± 0.81	**8.04 ± 0.51**	7.41 ± 0.51	**31.39 ± 2.46**	14.5 ± 0.90
	18:1	522.4	1.22 ± 0.07	1.40 ± 0.09	3.55 ± 0.26	4.52 ± 0.33	5.20 ± 0.42	5.34 ± 0.42	13.66 ± 1.26	7.31 ± 0.45
	**18:0**	**524.3**	2.67 ± 0.20	4.08 ± 0.31	3.59 ± 0.33	3.72 ± 0.37	**7.97 ± 0.51**	7.41 ± 0.46	**34.87 ± 2.15**	17.45 ± 0.91
	**20:4**	**544.3**	2.53 ± 0.16	2.27 ± 0.18	5.22 ± 0.52	6.00 ± 0.47	**4.36 ± 0.38**	3.90 ± 0.32	**15.63 ± 1.67**	7.16 ± 0.64
	**20:3**	**546.5**	**0.25 ± 0.02**	0.23 ± 0.02	**1.14 ± 0.08**	1.40 ± 0.10	**0.48 ± 0.04**	0.44 ± 0.03	**4.01 ± 0.41**	1.65 ± 0.14
	**22:6**	**568.4**	1.52 ± 0.13	1.61 ± 0.18	2.72 ± 0.21	2.75 ± 0.21	**2.93 ± 0.21**	3.75 ± 0.20	**12.06 ± 1.27**	4.68 ± 0.42
	**22:5**	**570.3**	0.47 ± 0.03	0.54 ± 0.04	1.33 ± 0.09	1.38 ± 0.08	**0.89 ± 0.08**	0.91 ± 0.06	**3.20 ± 0.22**	1.43 ± 0.10
	**22:4**	**572.4**	0.06 ± 0.00	0.06 ± 0.00	0.13 ± 0.01	0.19 ± 0.01	**0.14 ± 0.01**	0.12 ± 0.01	**0.48 ± 0.05**	0.21 ± 0.02
**PC**	**30:0**	**706.5**	0.33 ± 0.03	0.37 ± 0.05	0.33 ± 0.05	0.59 ± 0.06	**0.61 ± 0.06**	1.37 ± 0.08	**0.19 ± 0.02**	0.46 ± 0.04
	32:2	730.6	0.96 ± 0.10	1.28 ± 0.13	1.17 ± 0.12	2.56 ± 0.19	0.97 ± 0.09	1.75 ± 0.12	0.43 ± 0.04	1.27 ± 0.09
	32:1	732.5	1.27 ± 0.12	2.03 ± 0.16	2.79 ± 0.35	6.02 ± 0.82	1.49 ± 0.16	4.10 ± 0.35	1.26 ± 0.09	3.38 ± 0.24
	32:0	734.5	7.29 ± 0.52	8.95 ± 0.80	4.79 ± 0.65	8.60 ± 1.26	7.64 ± 0.46	16.72 ± 0.81	3.43 ± 0.30	7.12 ± 0.66
	34:4	754.5	1.14 ± 0.09	1.27 ± 0.10	0.51 ± 0.05	1.05 ± 0.09	0.64 ± 0.05	1.21 ± 0.08	0.32 ± 0.03	0.81 ± 0.06
	34:3	756.5	3.78 ± 0.53	5.05 ± 0.61	5.85 ± 0.75	12.08 ± 1.01	3.36 ± 0.30	5.79 ± 0.43	2.20 ± 0.20	6.58 ± 0.50
	34:2	758.6	54.20 ± 4.89	84.17 ± 7.39	58.09 ± 7.84	98.26 ± 8.83	53.06 ± 4.93	127.63 ± 7.50	34.16 ± 1.99	67.24 ± 4.40

class	acyl chain	*m/z*	gastrocnemius				soleus			
			C	CX	D	DX	C	CX	D	DX
	34:1	760.5	40.07 ± 2.74	57.45 ± 4.67	32.37 ± 2.04	63.20 ± 4.09	40.12 ± 4.10	104.35 ± 5.36	23.91 ± 1.50	50.91 ± 3.50
	**34:0**	**762.5**	**6.02 ± 0.60**	7.19 ± 0.60	**1.57 ± 0.22**	3.31 ± 0.27	**5.52 ± 0.26**	8.94 ± 0.85	**1.49 ± 0.13**	3.95 ± 0.30
	36:5[a]	780.5	2.17 ± 0.26	3.04 ± 0.28	1.12 ± 0.15	2.02 ± 0.26	1.12 ± 0.07	1.80 ± 0.11	0.55 ± 0.07	3.24 ± 0.19[*]
	36:5[b]	780.6	2.53 ± 0.23	2.98 ± 0.31	1.96 ± 0.26	4.08 ± 0.31	1.31 ± 0.13	2.34 ± 0.16	1.24 ± 0.16	3.35 ± 0.34
	36:5[c]	780.5	2.42 ± 0.22	2.90 ± 0.28	1.28 ± 0.32	1.80 ± 0.15	1.64 ± 0.08	3.63 ± 0.20	0.86 ± 0.16	2.19 ± 0.48
	36:4[a]	782.5	38.28 ± 3.81	47.40 ± 7.06	18.63 ± 0.69	33.80 ± 1.74	21.73 ± 3.35	48.07 ± 2.89	14.54 ± 0.89	33.95 ± 2.67
	36:4[b]	782.5	38.36 ± 6.13	39.69 ± 6.33	23.22 ± 4.25	37.46 ± 4.02	21.25 ± 1.69	36.62 ± 2.57	13.76 ± 1.07	33.00 ± 2.53
	36:3	784.5	20.88 ± 1.47	30.16 ± 2.84	21.96 ± 2.30	35.62 ± 3.41	21.16 ± 2.39	64.62 ± 5.06	18.15 ± 1.17	36.13 ± 3.55
	36:2	786.5	34.90 ± 2.91	55.83 ± 4.30	24.41 ± 1.86	47.62 ± 3.70	59.73 ± 2.10	116.96 ± 12.35	35.47 ± 2.56	77.11 ± 5.39
	36:1	788.5	16.75 ± 1.87	24.28 ± 2.59	6.39 ± 0.79	15.00 ± 1.31	21.56 ± 1.15	37.37 ± 3.02	10.43 ± 0.95	29.55 ± 2.07
	**36:0**	**790.6**	**0.56 ± 0.11**	0.84 ± 0.19	**0.12 ± 0.02**	0.36 ± 0.05	**3.02 ± 0.43**	6.00 ± 0.99	**0.79 ± 0.10**	1.43 ± 0.14
	38:7	804.5	4.60 ± 0.70	6.86 ± 1.01	1.96 ± 0.31	3.49 ± 0.37	3.50 ± 0.27	10.33 ± 0.47	2.19 ± 0.26	6.53 ± 0.51
	38:6[a]	806.6	19.35 ± 2.46	27.61 ± 4.51	15.14 ± 0.84	24.57 ± 1.56	20.95 ± 1.43	36.05 ± 4.27	11.52 ± 0.96	28.75 ± 2.33
	38:6[b]	806.5	20.47 ± 3.25	23.95 ± 4.89	10.23 ± 1.95	16.29 ± 1.39	21.04 ± 1.76	35.87 ± 3.57	10.82 ± 1.14	23.79 ± 2.60
	38:5	808.5	17.17 ± 1.79	25.91 ± 2.52	12.11 ± 1.69	17.73 ± 1.88	20.73 ± 1.23	33.32 ± 1.53	9.28 ± 0.83	19.26 ± 1.90
	38:4	810.5	32.45 ± 2.76	43.77 ± 3.24	13.13 ± 1.64	20.48 ± 1.68	36.35 ± 2.08	81.87 ± 6.20	19.58 ± 1.49	44.18 ± 2.74
	38:3	812.5	9.05 ± 0.73	13.01 ± 0.97	4.83 ± 0.53	8.39 ± 0.40	11.16 ± 0.42	19.17 ± 1.83	9.97 ± 1.15	19.82 ± 1.20
	**38:2**	**814.6**	**5.14 ± 0.77**	7.06 ± 0.75	**1.13 ± 0.15**	3.26 ± 0.27	0.86 ± 0.17	4.93 ± 0.27	1.72 ± 0.24	4.88 ± 0.45
	**40:8**	**830.5**	**7.57 ± 0.67**	10.39 ± 0.86	**1.98 ± 0.27**	3.03 ± 0.25	19.00 ± 1.48	38.13 ± 2.30	7.94 ± 0.88	20.59 ± 2.34
	40:7	832.6	4.39 ± 0.49	6.18 ± 0.72	3.42 ± 0.57	4.50 ± 0.34	8.14 ± 0.69	16.88 ± 1.21	3.63 ± 0.33	8.50 ± 0.90
	40:6	834.5	7.91 ± 0.77	11.50 ± 1.11	6.75 ± 0.95	10.15 ± 0.93	16.98 ± 1.41	34.46 ± 3.77	6.73 ± 0.82	15.83 ± 1.69
	40:5	836.5	3.71 ± 0.35	5.46 ± 0.43	2.88 ± 0.38	4.61 ± 0.35	6.85 ± 0.34	12.93 ± 0.86	2.48 ± 0.31	6.14 ± 0.54
	40:4	838.5	1.90 ± 0.14	2.94 ± 0.25	1.35 ± 0.16	1.90 ± 0.15	1.48 ± 0.08	4.09 ± 0.27	0.91 ± 0.16	2.66 ± 0.32
	40:1	844.5	0.50 ± 0.08	0.72 ± 0.11	0.34 ± 0.07	0.46 ± 0.06	1.29 ± 0.15	6.51 ± 0.80[*]	0.43 ± 0.08	1.81 ± 0.21[*]

class	acyl chain	*m/z*	gastrocnemius				soleus			
			C	CX	D	DX	C	CX	D	DX
	40:0	846.6	0.11 ± 0.02	0.08 ± 0.01	0.07 ± 0.02	0.06 ± 0.01	0.40 ± 0.05	0.74 ± 0.07	0.14 ± 0.02	0.38 ± 0.02
	42:10	854.5	0.97 ± 0.09	0.82 ± 0.06	0.54 ± 0.08	0.46 ± 0.03	2.39 ± 0.20	5.32 ± 0.36	1.24 ± 0.20	1.79 ± 0.13
	**42:1**	**872.5**	0.43 ± 0.08	0.49 ± 0.09	0.28 ± 0.07	0.43 ± 0.05	**1.12 ± 0.18**	4.31 ± 0.76[*]	**0.36 ± 0.07**	1.67 ± 0.20[*]
**LPE**	**16:1**	**452.4**	**0.01 ± 0.00**	0.01 ± 0.00	**0.05 ± 0.00**	0.06 ± 0.00	0.02 ± 0.00	0.02 ± 0.00	0.03 ± 0.00	0.02 ± 0.00
	16:0	454.3	0.19 ± 0.01	0.16 ± 0.02	0.30 ± 0.02	0.32 ± 0.03	0.23 ± 0.03	0.25 ± 0.03	0.34 ± 0.04	0.28 ± 0.02
	18:2	478.4	0.20 ± 0.02	0.16 ± 0.01	0.35 ± 0.03	0.42 ± 0.03	0.44 ± 0.03	0.37 ± 0.02	0.41 ± 0.05	0.34 ± 0.02
	18:1	480.3	0.26 ± 0.03	0.38 ± 0.07	0.53 ± 0.04	0.65 ± 0.05	0.48 ± 0.03	0.53 ± 0.12	0.65 ± 0.06	0.32 ± 0.02
	18:0	482.5	0.37 ± 0.04	0.25 ± 0.02	0.52 ± 0.05	0.58 ± 0.06	1.04 ± 0.08	0.88 ± 0.06	1.29 ± 0.07	0.70 ± 0.05
	**20:5**	**500.3**	**0.01 ± 0.00**	0.01 ± 0.00	**0.03 ± 0.00**	0.03 ± 0.00	**0.02 ± 0.00**	0.02 ± 0.00	**0.07 ± 0.00**	0.02 ± 0.00[*]
	20:4	502.4	0.25 ± 0.02	0.19 ± 0.02	0.33 ± 0.02	0.28 ± 0.02	0.37 ± 0.03	0.26 ± 0.02	1.10 ± 0.13	0.58 ± 0.04
	20:3	504.3	0.02 ± 0.00	0.01 ± 0.00	0.03 ± 0.00	0.03 ± 0.00	0.03 ± 0.00	0.02 ± 0.00	0.09 ± 0.01	0.05 ± 0.01
	22:6	526.5	1.29 ± 0.11	1.11 ± 0.11	1.61 ± 0.12	1.27 ± 0.11	1.34 ± 0.11	0.92 ± 0.08	2.59 ± 0.14	1.78 ± 0.10
	**22:5**	**528.4**	0.44 ± 0.05	0.30 ± 0.04	0.59 ± 0.06	0.53 ± 0.05	**0.44 ± 0.04**	0.24 ± 0.02	**1.46 ± 0.11**	0.64 ± 0.06
	**22:4**	**530.4**	0.11 ± 0.01	0.07 ± 0.01	0.11 ± 0.01	0.11 ± 0.01	**0.09 ± 0.01**	0.07 ± 0.01	**0.28 ± 0.02**	0.19 ± 0.02
**PE**	34:3	714.5	0.01 ± 0.00	0.02 ± 0.00	0.02 ± 0.00	0.04 ± 0.00	0.01 ± 0.00	0.02 ± 0.00	0.01 ± 0.00	0.05 ± 0.00[*]
	34:2	716.5	0.32 ± 0.03	0.72 ± 0.08	0.29 ± 0.04	0.78 ± 0.08	0.28 ± 0.03	0.70 ± 0.05	0.23 ± 0.02	0.81 ± 0.07[*]
	34:1	718.5	0.11 ± 0.01	0.28 ± 0.04	0.09 ± 0.01	0.32 ± 0.03[*]	0.11 ± 0.01	0.30 ± 0.02	0.22 ± 0.02	0.43 ± 0.03
	**34:0**	**720.6**	**0.05 ± 0.01**	0.09 ± 0.01	**0.01 ± 0.00**	0.03 ± 0.00	0.05 ± 0.00	0.12 ± 0.01	0.04 ± 0.00	0.11 ± 0.01
	36:5	738.5	0.07 ± 0.01	0.17 ± 0.02	0.07 ± 0.01	0.15 ± 0.01	0.07 ± 0.01	0.22 ± 0.01[*]	0.02 ± 0.00	0.06 ± 0.00
	36:4[a]	740.5	0.41 ± 0.05	0.61 ± 0.07	0.14 ± 0.02	0.42 ± 0.06	0.37 ± 0.02	0.29 ± 0.02	0.18 ± 0.01	0.72 ± 0.06[*]
	36:4[b]	740.5	0.24 ± 0.04	0.68 ± 0.07	0.21 ± 0.03	0.39 ± 0.07	0.19 ± 0.02	0.34 ± 0.03	0.20 ± 0.01	0.60 ± 0.05[*]
	36:3	742.6	0.35 ± 0.04	0.80 ± 0.09	0.28 ± 0.04	0.66 ± 0.05	0.42 ± 0.03	1.09 ± 0.06	0.26 ± 0.02	0.92 ± 0.09[*]
	36:2	744.5	2.57 ± 0.27	5.97 ± 0.57	1.65 ± 0.24	4.50 ± 0.39	1.60 ± 0.20	6.47 ± 0.35	1.94 ± 0.18	4.21 ± 0.43
	36:1	746.5	0.43 ± 0.05	0.85 ± 0.07	0.19 ± 0.03	0.54 ± 0.05	0.29 ± 0.02	0.71 ± 0.06	0.49 ± 0.04	0.81 ± 0.07

class	acyl chain	*m/z*	gastrocnemius C	gastrocnemius CX	gastrocnemius D	gastrocnemius DX	soleus C	soleus CX	soleus D	soleus DX
	38:7	762.5	0.04 ± 0.00	0.08 ± 0.01	0.06 ± 0.01	0.14 ± 0.02	0.02 ± 0.00	0.05 ± 0.00	0.04 ± 0.00	0.14 ± 0.01*
	38:6[a]	764.5	0.53 ± 0.09	1.27 ± 0.17	0.49 ± 0.10	0.94 ± 0.18	0.62 ± 0.05	1.37 ± 0.08	0.36 ± 0.04	1.20 ± 0.14*
	38:6[b]	764.6	0.50 ± 0.07	0.67 ± 0.09	0.49 ± 0.05	0.58 ± 0.08	0.53 ± 0.03	0.81 ± 0.05	0.24 ± 0.02	0.98 ± 0.05*
	38:5	766.5	0.63 ± 0.06	1.29 ± 0.13	0.62 ± 0.12	0.94 ± 0.09	0.69 ± 0.07	0.95 ± 0.06	0.38 ± 0.04	1.21 ± 0.13*
	38:4	768.5	4.24 ± 0.39	7.70 ± 0.96	2.08 ± 0.34	3.53 ± 0.27	3.23 ± 0.27	8.26 ± 0.31	2.77 ± 0.15	7.21 ± 0.64
	38:3	770.6	0.72 ± 0.06	1.33 ± 0.14	0.53 ± 0.08	0.86 ± 0.06	0.70 ± 0.05	0.98 ± 0.07	0.41 ± 0.07	1.43 ± 0.14*
	38:2	772.5	0.07 ± 0.01	0.17 ± 0.02	0.04 ± 0.01	0.10 ± 0.01	0.07 ± 0.00	0.13 ± 0.01	0.10 ± 0.00	0.30 ± 0.02
	40:8	788.6	1.34 ± 0.18	2.09 ± 0.33	0.77 ± 0.17	0.99 ± 0.14	0.73 ± 0.15	1.25 ± 0.23	0.52 ± 0.10	1.53 ± 0.26
	40:7	790.5	0.65 ± 0.07	1.50 ± 0.16	0.69 ± 0.12	0.88 ± 0.11	0.94 ± 0.06	1.90 ± 0.20	0.45 ± 0.05	1.27 ± 0.11
	40:6	792.6	5.76 ± 0.51	13.56 ± 1.46	3.40 ± 0.57	6.17 ± 0.47	6.36 ± 0.49	11.39 ± 0.88	3.42 ± 0.20	9.72 ± 1.07
	40:5	794.5	1.96 ± 0.14	3.99 ± 0.42	1.30 ± 0.21	2.36 ± 0.17	1.07 ± 0.09	3.21 ± 0.18	1.41 ± 0.17	2.81 ± 0.35
	40:4	796.6	0.62 ± 0.05	1.15 ± 0.10	0.42 ± 0.07	0.69 ± 0.05	0.47 ± 0.02	1.09 ± 0.08	0.35 ± 0.02	0.85 ± 0.08
	**40:3**	**798.6**	0.04 ± 0.01	0.08 ± 0.01	0.01 ± 0.00	0.03 ± 0.00	**0.01 ± 0.00**	0.11 ± 0.01*	**0.05 ± 0.00**	0.11 ± 0.02
**LPG**	**14:0**	**455.4**	**0.01 ± 0.00**	0.01 ± 0.00	**0.03 ± 0.00**	0.03 ± 0.00	0.03 ± 0.00	0.02 ± 0.00	0.06 ± 0.01	0.02 ± 0.00*
	**16:1**	**481.5**	**0.01 ± 0.00**	0.02 ± 0.00	**0.07 ± 0.01**	0.10 ± 0.01	**0.05 ± 0.01**	0.06 ± 0.00	**0.31 ± 0.02**	0.13 ± 0.01
	16:0	483.3	0.20 ± 0.04	0.17 ± 0.01	0.55 ± 0.12	0.59 ± 0.09	2.53 ± 0.27	1.55 ± 0.12	2.69 ± 0.30	0.71 ± 0.06*
	18:2	507.5	0.37 ± 0.04	0.46 ± 0.05	0.80 ± 0.08	0.91 ± 0.08	3.03 ± 0.36	1.71 ± 0.20	3.40 ± 0.41	0.73 ± 0.09*
	18:1	509.4	0.52 ± 0.10	0.58 ± 0.06	0.95 ± 0.16	1.12 ± 0.16	3.06 ± 0.31	2.19 ± 0.22	4.27 ± 0.68	1.10 ± 0.10*
	18:0	511.4	0.06 ± 0.01	0.07 ± 0.01	0.09 ± 0.02	0.10 ± 0.02	0.34 ± 0.04	0.45 ± 0.03	0.68 ± 0.09	0.18 ± 0.02*
**PG**	16:1/16:0	719.5	0.06 ± 0.01	0.08 ± 0.01	0.09 ± 0.01	0.14 ± 0.01	0.04 ± 0.01	0.03 ± 0.00	0.03 ± 0.00	0.04 ± 0.00
	18:1/14:0	719.5	0.04 ± 0.01	0.05 ± 0.01	0.06 ± 0.01	0.08 ± 0.01	0.04 ± 0.01	0.04 ± 0.00	0.03 ± 0.00	0.04 ± 0.00
	**16:0/16:0**	**721.6**	0.35 ± 0.05	0.31 ± 0.04	0.17 ± 0.02	0.26 ± 0.04	**0.20 ± 0.02**	0.10 ± 0.01	**0.06 ± 0.01**	0.08 ± 0.01
	18:1/16:1	745.5	0.25 ± 0.03	0.28 ± 0.02	0.32 ± 0.03	0.31 ± 0.02	0.35 ± 0.03	0.29 ± 0.02	0.31 ± 0.03	0.34 ± 0.04
	18:2/16:0	745.5	1.89 ± 0.36	3.70 ± 0.93	1.53 ± 0.10	2.03 ± 0.17	1.13 ± 0.26	0.99 ± 0.28	0.52 ± 0.09	0.55 ± 0.05

class	acyl chain	*m/z*	gastrocnemius				soleus			
			C	CX	D	DX	C	CX	D	DX
	18:1/16:0	747.5	6.07 ± 0.87	6.64 ± 0.88	4.02 ± 0.44	6.82 ± 1.15	3.14 ± 0.43	2.03 ± 0.29	1.31 ± 0.27	1.93 ± 0.36
	18:2/18:2	769.6	0.44 ± 0.06	0.64 ± 0.14	0.30 ± 0.02	0.31 ± 0.03	0.27 ± 0.06	0.16 ± 0.04	0.10 ± 0.01	0.09 ± 0.01
	**20:4/16:0**	**769.6**	0.08 ± 0.01	0.09 ± 0.02	0.03 ± 0.00	0.03 ± 0.00	**0.05 ± 0.01**	0.03 ± 0.01	**0.02 ± 0.00**	0.01 ± 0.00
	18:2/18:1	771.6	0.38 ± 0.06	0.41 ± 0.07	0.42 ± 0.03	0.40 ± 0.05	0.18 ± 0.03	0.12 ± 0.02	0.13 ± 0.03	0.11 ± 0.02
	18:1/18:1	773.6	0.18 ± 0.02	0.21 ± 0.02	0.22 ± 0.03	0.17 ± 0.03	0.18 ± 0.03	0.16 ± 0.02	0.15 ± 0.03	0.18 ± 0.01
	**18:2/18:0**	**773.6**	1.07 ± 0.12	1.25 ± 0.20	0.43 ± 0.06	0.70 ± 0.10	**0.57 ± 0.06**	0.37 ± 0.06	**0.15 ± 0.03**	0.20 ± 0.03
	20:2/16:0	773.6	0.32 ± 0.03	0.31 ± 0.02	0.41 ± 0.03	0.35 ± 0.03	0.28 ± 0.03	0.27 ± 0.02	0.24 ± 0.03	0.21 ± 0.01
	18:2/22:6	817.6	0.21 ± 0.03	0.22 ± 0.03	0.17 ± 0.01	0.14 ± 0.02	0.11 ± 0.01	0.07 ± 0.01	0.06 ± 0.01	0.04 ± 0.00
	18:1/22:6	819.5	N.Q.	N.Q.	N.Q.	N.Q.	N.Q.	N.Q.	N.Q.	N.Q.
	**22:6/22:6**	**865.6**	**0.07 ± 0.01**	0.08 ± 0.01	**0.02 ± 0.00**	0.02 ± 0.00	**0.05 ± 0.01**	0.03 ± 0.00	**0.01 ± 0.00**	0.01 ± 0.00
**LPI**	16:0	571.4	0.05 ± 0.01	0.07 ± 0.01	0.14 ± 0.02	0.18 ± 0.03	0.34 ± 0.03	0.24 ± 0.02	0.70 ± 0.14	0.50 ± 0.09
	**18:2**	**595.4**	**0.06 ± 0.01**	0.07 ± 0.01	**0.21 ± 0.02**	0.23 ± 0.03	0.35 ± 0.03	0.22 ± 0.02	1.06 ± 0.11	0.62 ± 0.09
	18:1	597.5	0.05 ± 0.01	0.06 ± 0.01	0.14 ± 0.02	0.19 ± 0.03	0.37 ± 0.05	0.22 ± 0.02	0.73 ± 0.14	0.53 ± 0.10
	18:0	599.3	0.40 ± 0.06	0.42 ± 0.06	0.91 ± 0.21	0.83 ± 0.16	2.01 ± 0.31	1.05 ± 0.19	4.12 ± 1.17	1.99 ± 0.40
	20:4	619.5	0.41 ± 0.04	0.39 ± 0.05	0.88 ± 0.10	0.83 ± 0.09	1.94 ± 0.19	1.00 ± 0.09	3.83 ± 0.42	2.11 ± 0.33
	**20:3**	**621.4**	**0.09 ± 0.03**	0.07 ± 0.01	**0.40 ± 0.05**	0.42 ± 0.05	**0.54 ± 0.19**	0.24 ± 0.02	**1.94 ± 0.26**	1.13 ± 0.16
	22:6	643.4	0.01 ± 0.00	0.01 ± 0.00	0.02 ± 0.00	0.02 ± 0.00	0.13 ± 0.01	0.08 ± 0.00	0.16 ± 0.01	0.11 ± 0.01
**PI**	16:0/16:0	809.6	0.03 ± 0.00	0.03 ± 0.01	0.03 ± 0.00	0.04 ± 0.01	0.10 ± 0.01	0.07 ± 0.00	0.10 ± 0.01	0.08 ± 0.01
	18:1/16:1	833.5	0.03 ± 0.00	0.03 ± 0.00	0.04 ± 0.00	0.03 ± 0.00	0.05 ± 0.01	0.06 ± 0.01	0.05 ± 0.01	0.07 ± 0.02
	18:2/16:0	833.5	0.05 ± 0.00	0.06 ± 0.01	0.04 ± 0.00	0.05 ± 0.00	0.12 ± 0.01	0.08 ± 0.01	0.11 ± 0.01	0.08 ± 0.01
	18:1/16:0	835.5	0.11 ± 0.01	0.13 ± 0.01	0.11 ± 0.01	0.14 ± 0.02	0.16 ± 0.01	0.11 ± 0.01	0.13 ± 0.01	0.12 ± 0.01
	16:0/20:4	857.6	0.16 ± 0.02	0.18 ± 0.02	0.14 ± 0.01	0.15 ± 0.01	0.19 ± 0.02	0.14 ± 0.01	0.15 ± 0.01	0.12 ± 0.01
	16:0/20:3	859.5	0.02 ± 0.00	0.04 ± 0.01	0.03 ± 0.01	0.04 ± 0.00	0.05 ± 0.01	0.04 ± 0.01	0.05 ± 0.01	0.06 ± 0.01
	18:1/18:2	859.5	0.07 ± 0.01	0.09 ± 0.01	0.06 ± 0.00	0.06 ± 0.00	0.13 ± 0.01	0.09 ± 0.01	0.11 ± 0.01	0.09 ± 0.01

class	acyl chain	*m/z*	gastrocnemius				soleus			
			C	CX	D	DX	C	CX	D	DX
	18:0/18:2	861.6	0.17 ± 0.03	0.23 ± 0.02	0.23 ± 0.02	0.25 ± 0.03	0.20 ± 0.02	0.15 ± 0.01	0.19 ± 0.02	0.16 ± 0.02
	18:1/18:1	861.6	0.08 ± 0.01	0.07 ± 0.02	0.06 ± 0.01	0.05 ± 0.01	0.07 ± 0.01	0.09 ± 0.02	0.08 ± 0.01	0.07 ± 0.01
	18:0/18:1	863.5	0.05 ± 0.01	0.04 ± 0.01	0.05 ± 0.00	0.07 ± 0.01	0.12 ± 0.01	0.07 ± 0.00	0.11 ± 0.01	0.09 ± 0.01
	**18:2/20:4**	**881.5**	**0.02 ± 0.00**	0.02 ± 0.00	**0.01 ± 0.00**	0.01 ± 0.00	0.10 ± 0.01	0.07 ± 0.00	0.09 ± 0.01	0.07 ± 0.01
	16:0/22:6	881.6	0.04 ± 0.00	0.05 ± 0.01	0.03 ± 0.00	0.04 ± 0.00	0.11 ± 0.01	0.08 ± 0.00	0.10 ± 0.01	0.08 ± 0.01
	16:0/22:5	883.5	0.04 ± 0.00	0.06 ± 0.01	0.05 ± 0.01	0.06 ± 0.01	0.06 ± 0.00	0.05 ± 0.01	0.05 ± 0.01	0.04 ± 0.00
	18:0/20:5	883.5	0.08 ± 0.01	0.09 ± 0.02	0.07 ± 0.01	0.08 ± 0.02	0.05 ± 0.01	0.04 ± 0.01	0.05 ± 0.00	0.04 ± 0.00
	18:1/20:4	883.5	0.31 ± 0.03	0.32 ± 0.04	0.18 ± 0.01	0.18 ± 0.02	0.30 ± 0.03	0.19 ± 0.02	0.16 ± 0.02	0.14 ± 0.01
	18:0/20:4	885.6	5.67 ± 0.65	4.57 ± 0.46	2.98 ± 0.25	2.86 ± 0.44	4.14 ± 0.43	1.98 ± 0.23	1.39 ± 0.28	1.22 ± 0.22
	18:0/20:3	887.5	0.36 ± 0.05	0.28 ± 0.05	0.55 ± 0.06	0.48 ± 0.09	0.38 ± 0.05	0.18 ± 0.03	0.34 ± 0.06	0.25 ± 0.04
	18:0/20:2	889.5	N.Q.	N.Q.	N.Q.	N.Q.	N.Q.	N.Q.	N.Q.	N.Q.
	18:1/22:6	907.5	0.04 ± 0.00	0.04 ± 0.01	0.02 ± 0.00	0.02 ± 0.00	0.11 ± 0.01	0.07 ± 0.00	0.09 ± 0.01	0.07 ± 0.01
	18:0/22:6	909.6	0.55 ± 0.08	0.55 ± 0.06	0.38 ± 0.03	0.34 ± 0.05	0.47 ± 0.06	0.29 ± 0.03	0.24 ± 0.04	0.20 ± 0.02
	18:0/22:5	911.5	0.24 ± 0.03	0.23 ± 0.03	0.21 ± 0.02	0.21 ± 0.04	0.25 ± 0.02	0.15 ± 0.02	0.18 ± 0.03	0.15 ± 0.02
	18:0/22:4	913.6	0.09 ± 0.01	0.16 ± 0.03	0.06 ± 0.01	0.07 ± 0.02	0.16 ± 0.01	0.12 ± 0.01	0.11 ± 0.01	0.09 ± 0.01
**LPS**	**18:1**	**522.3**	**0.06 ± 0.01**	0.10 ± 0.01	**0.24 ± 0.04**	0.37 ± 0.06	**0.23 ± 0.03**	0.16 ± 0.02	**0.70 ± 0.16**	0.60 ± 0.12
	22:6	568.4	0.26 ± 0.02	0.29 ± 0.03	0.71 ± 0.08	0.75 ± 0.08	0.77 ± 0.07	0.47 ± 0.04	1.84 ± 0.25	1.19 ± 0.14
**PS**	16:0/18:2	758.6	0.24 ± 0.03	0.36 ± 0.05	0.69 ± 0.07	0.56 ± 0.05	0.19 ± 0.03	0.18 ± 0.03	0.33 ± 0.07	0.22 ± 0.03
	16:1/18:1	758.6	0.08 ± 0.02	0.09 ± 0.02	0.07 ± 0.01	0.08 ± 0.01	0.05 ± 0.01	0.07 ± 0.01	0.06 ± 0.01	0.05 ± 0.01
	16:0/18:1	760.5	0.02 ± 0.00	0.03 ± 0.00	0.02 ± 0.01	0.03 ± 0.01	0.03 ± 0.00	0.02 ± 0.00	0.02 ± 0.00	0.02 ± 0.00
	18:0/16:1	760.5	0.01 ± 0.00	0.01 ± 0.00	0.01 ± 0.01	0.01 ± 0.00	0.00 ± 0.00	0.00 ± 0.00	0.00 ± 0.00	0.00 ± 0.00
	16:0/20:4	782.5	N.Q.	N.Q.	N.Q.	N.Q.	N.Q.	N.Q.	N.Q.	N.Q.
	18:1/18:2	784.5	0.05 ± 0.01	0.12 ± 0.02	0.05 ± 0.01	0.05 ± 0.00	0.04 ± 0.00	0.06 ± 0.01	0.03 ± 0.00	0.03 ± 0.00
	18:0/18:2	786.6	0.14 ± 0.02	0.10 ± 0.01	0.15 ± 0.02	0.12 ± 0.01	0.08 ± 0.02	0.07 ± 0.01	0.06 ± 0.01	0.05 ± 0.01

class	acyl chain	*m/z*	gastrocnemius				soleus			
			C	CX	D	DX	C	CX	D	DX
	18:1/18:1	786.6	0.26 ± 0.03	0.46 ± 0.03	0.25 ± 0.04	0.36 ± 0.05	0.20 ± 0.02	0.18 ± 0.02	0.11 ± 0.02	0.16 ± 0.03
	18:0/18:1	788.5	0.65 ± 0.09	0.92 ± 0.17	0.54 ± 0.19	0.85 ± 0.15	0.46 ± 0.09	0.39 ± 0.09	0.25 ± 0.07	0.36 ± 0.08
	**16:0/22:6**	**806.5**	0.21 ± 0.03	0.26 ± 0.04	0.09 ± 0.02	0.12 ± 0.01	**0.17 ± 0.03**	0.12 ± 0.02	**0.05 ± 0.01**	0.06 ± 0.01
	18:1/20:4	808.5	0.06 ± 0.01	0.09 ± 0.01	0.05 ± 0.01	0.05 ± 0.00	0.06 ± 0.01	0.04 ± 0.01	0.03 ± 0.01	0.03 ± 0.00
	16:0/22:5	808.5	N.Q.	N.Q.	N.Q.	N.Q.	N.Q.	N.Q.	N.Q.	N.Q.
	**18:0/20:4**	**810.6**	0.71 ± 0.08	0.79 ± 0.05	0.40 ± 0.06	0.38 ± 0.05	**0.52 ± 0.06**	0.35 ± 0.03	**0.17 ± 0.03**	0.16 ± 0.03
	18:0/20:3	812.5	0.14 ± 0.02	0.14 ± 0.02	0.14 ± 0.02	0.13 ± 0.02	0.10 ± 0.01	0.07 ± 0.01	0.07 ± 0.01	0.06 ± 0.01
	20:0/18:1	816.5	0.10 ± 0.02	0.27 ± 0.07	0.10 ± 0.03	0.11 ± 0.02	N.Q.	N.Q.	N.Q.	N.Q.
	**18:1/22:6**	**832.6**	0.17 ± 0.03	0.22 ± 0.04	0.08 ± 0.01	0.07 ± 0.01	**0.13 ± 0.02**	0.10 ± 0.02	**0.04 ± 0.01**	0.04 ± 0.00
	**18:0/22:6**	**834.5**	5.62 ± 0.49	5.06 ± 0.47	2.86 ± 0.36	2.73 ± 0.31	**4.11 ± 0.38**	2.18 ± 0.26	**1.23 ± 0.22**	1.17 ± 0.18
	18:0/22:5	836.6	1.01 ± 0.11	0.96 ± 0.08	0.64 ± 0.10	0.64 ± 0.09	0.69 ± 0.07	0.43 ± 0.05	0.27 ± 0.06	0.29 ± 0.05
	18:0/22:4	838.5	0.35 ± 0.05	0.40 ± 0.06	0.21 ± 0.02	0.19 ± 0.03	0.24 ± 0.03	0.19 ± 0.03	0.10 ± 0.01	0.09 ± 0.02
	22:1/18:1	842.6	0.05 ± 0.01	0.18 ± 0.04	0.05 ± 0.02	0.06 ± 0.02	0.04 ± 0.01	0.07 ± 0.02	0.03 ± 0.01	0.03 ± 0.01
	22:0/18:1	844.6	0.13 ± 0.04	0.48 ± 0.16	0.19 ± 0.08	0.22 ± 0.10	N.Q.	N.Q.	N.Q.	N.Q.
	24:1/18:1	870.5	N.Q.	N.Q.	N.Q.	N.Q.	N.Q.	N.Q.	N.Q.	N.Q.
	24:0/18:1	872.5	N.Q.	N.Q.	N.Q.	N.Q.	N.Q.	N.Q.	N.Q.	N.Q.
**LPA**	16:0	409.4	0.02 ± 0.00	0.02 ± 0.01	0.05 ± 0.01	0.04 ± 0.01	0.07 ± 0.01	0.04 ± 0.00	0.10 ± 0.01	0.08 ± 0.01
	**18:2**	**433.5**	**0.01 ± 0.00**	0.01 ± 0.00	**0.03 ± 0.01**	0.02 ± 0.00	0.05 ± 0.00	0.05 ± 0.00	0.09 ± 0.01	0.06 ± 0.01
	18:0	437.3	0.01 ± 0.00	0.01 ± 0.00	0.03 ± 0.01	0.01 ± 0.00	0.04 ± 0.01	0.02 ± 0.00	0.05 ± 0.01	0.03 ± 0.00
**PA**	**16:0/18:2**	**671.5**	**0.03 ± 0.00**	0.03 ± 0.00	**0.11 ± 0.03**	0.06 ± 0.02	0.04 ± 0.00	0.02 ± 0.00	0.01 ± 0.00	0.01 ± 0.00
	16:1/18:1	671.5	0.02 ± 0.00	0.02 ± 0.00	0.02 ± 0.00	0.03 ± 0.01	0.03 ± 0.00	0.03 ± 0.00	0.02 ± 0.00	0.04 ± 0.01
**SM**	d18:1/16:0	703.5	0.54 ± 0.07	0.59 ± 0.04	0.58 ± 0.07	0.58 ± 0.07	1.51 ± 0.08	1.59 ± 0.16	4.09 ± 0.34	1.14 ± 0.10*
	d18:1/18:0	731.6	12.01 ± 1.37	10.89 ± 0.98	6.62 ± 0.80	6.94 ± 0.78	2.46 ± 0.13	2.05 ± 0.20	5.86 ± 0.82	2.13 ± 0.14
	**d18:1/20:0**	**759.5**	**3.21 ± 0.38**	3.26 ± 0.31	**1.01 ± 0.12**	1.32 ± 0.21	2.54 ± 0.12	2.27 ± 0.20	7.57 ± 0.49	3.27 ± 0.22

class	acyl chain	*m/z*	gastrocnemius				soleus			
			C	CX	D	DX	C	CX	D	DX
	d18:1/22:1	785.5	0.80 ± 0.08	0.91 ± 0.07	0.27 ± 0.03	0.47 ± 0.09	0.27 ± 0.03	0.23 ± 0.02	0.27 ± 0.03	0.14 ± 0.02
	**d18:1/22:0**	**787.6**	**4.65 ± 0.68**	5.16 ± 0.92	**1.21 ± 0.17**	1.75 ± 0.16	1.52 ± 0.19	1.25 ± 0.18	1.20 ± 0.16	0.52 ± 0.06
	**d18:1/23:0**	**801.6**	**2.57 ± 0.42**	2.13 ± 0.29	**0.42 ± 0.06**	0.62 ± 0.11	0.90 ± 0.12	0.56 ± 0.07	0.44 ± 0.06	0.18 ± 0.03
	**d18:1/24:2**	**811.6**	**2.21 ± 0.36**	2.29 ± 0.56	**0.50 ± 0.05**	0.74 ± 0.04	0.72 ± 0.13	0.56 ± 0.10	0.48 ± 0.05	0.22 ± 0.01
	d18:1/24:1	813.5	4.57 ± 0.67	3.65 ± 0.77	2.92 ± 0.47	4.39 ± 0.43	13.09 ± 1.42	10.7 ± 1.30	27.24 ± 3.76	12.90 ± 1.43
	**d18:1/24:0**	**815.6**	**6.90 ± 0.86**	7.20 ± 0.93	**1.44 ± 0.24**	2.42 ± 0.26	27.04 ± 3.85	20.95 ± 2.07	16.51 ± 2.27	8.01 ± 0.96
**Cer**	**d18:1/16:0**	**538.6**	0.02 ± 0.00	0.02 ± 0.00	0.02 ± 0.00	0.03 ± 0.00	**0.01 ± 0.00**	0.01 ± 0.00	**0.03 ± 0.00**	0.01 ± 0.00*
	**d18:0/18:1**	**564.5**	0.00 ± 0.00	0.00 ± 0.00	0.00 ± 0.00	0.00 ± 0.00	**0.01 ± 0.00**	0.01 ± 0.00	**0.02 ± 0.00**	0.01 ± 0.00*
	d18:1/18:0	566.5	0.52 ± 0.04	0.55 ± 0.05	0.38 ± 0.04	0.41 ± 0.04	0.10 ± 0.01	0.09 ± 0.01	0.22 ± 0.02	0.07 ± 0.01*
	d18:1/20:0	594.8	N.Q.	N.Q.	N.Q.	N.Q.	N.Q.	N.Q.	N.Q.	N.Q.
	d18:1/22:0	622.5	0.09 ± 0.02	0.11 ± 0.01	0.03 ± 0.00	0.04 ± 0.00	0.02 ± 0.00	0.02 ± 0.00	0.03 ± 0.00	0.01 ± 0.00*
	d18:1/24:2	646.5	0.10 ± 0.01	0.12 ± 0.01	0.04 ± 0.00	0.06 ± 0.01	0.02 ± 0.00	0.02 ± 0.00	0.04 ± 0.00	0.01 ± 0.00
	**d18:1/24:1**	**648.6**	**0.61 ± 0.09**	0.74 ± 0.06	**0.20 ± 0.02**	0.32 ± 0.03	0.11 ± 0.01	0.11 ± 0.01	0.13 ± 0.01	0.05 ± 0.00
	**d18:1/24:0**	**650.6**	**0.98 ± 0.13**	1.12 ± 0.12	**0.22 ± 0.02**	0.44 ± 0.07	0.18 ± 0.02	0.16 ± 0.01	0.13 ± 0.01	0.07 ± 0.01
**MHC**	d18:1/24:1	810.6	0.18 ± 0.03	0.27 ± 0.02	0.10 ± 0.01	0.16 ± 0.01	0.07 ± 0.00	0.09 ± 0.01	0.13 ± 0.01	0.07 ± 0.01
	d18:1/24:0	812.5	N.Q.	N.Q.	N.Q.	N.Q.	N.Q.	N.Q.	N.Q.	N.Q.
**DAG**	14:1,16:1	554.5	N.Q.	N.Q.	N.Q.	N.Q.	N.Q.	N.Q.	N.Q.	N.Q.
	14:0,16:1	556.5	0.00 ± 0.00	0.00 ± 0.00	0.00 ± 0.00	0.00 ± 0.00	0.01 ± 0.00	0.01 ± 0.00	0.01 ± 0.01	0.01 ± 0.00
	**14:1,16:0**	**556.5**	**0.00 ± 0.00**	0.00 ± 0.00	**0.00 ± 0.00**	0.00 ± 0.00	**0.01 ± 0.00**	0.01 ± 0.00	**0.04 ± 0.01**	0.02 ± 0.00
	**14:0,16:0**	**558.5**	0.00 ± 0.00	0.00 ± 0.00	0.01 ± 0.00	0.00 ± 0.00	**0.01 ± 0.00**	0.01 ± 0.00	**0.02 ± 0.00**	0.01 ± 0.00
	14:0,18:2	582.6	0.00 ± 0.00	0.00 ± 0.00	0.01 ± 0.00	0.01 ± 0.00	0.03 ± 0.00	0.04 ± 0.01	0.05 ± 0.00	0.04 ± 0.01
	16:1,16:1	582.6	0.00 ± 0.00	0.00 ± 0.00	0.01 ± 0.00	0.01 ± 0.00	0.11 ± 0.02	0.11 ± 0.01	0.12 ± 0.02	0.10 ± 0.01
	14:0,18:1	584.5	0.00 ± 0.00	0.01 ± 0.00	0.01 ± 0.00	0.00 ± 0.00	0.03 ± 0.01	0.04 ± 0.00	0.05 ± 0.01	0.04 ± 0.00
	16:0,16:1	584.5	0.00 ± 0.00	0.00 ± 0.00	0.01 ± 0.00	0.00 ± 0.00	0.05 ± 0.01	0.04 ± 0.00	0.07 ± 0.01	0.05 ± 0.00

class	acyl chain	*m/z*	gastrocnemius				soleus			
			C	CX	D	DX	C	CX	D	DX
	16:0,16:0	586.6	0.22 ± 0.02	0.27 ± 0.02	0.22 ± 0.02	0.15 ± 0.01	0.05 ± 0.01	0.04 ± 0.00	0.09 ± 0.01	0.11 ± 0.02
	**16:1,18:3**	**606.5**	**0.00 ± 0.00**	0.00 ± 0.00	**0.00 ± 0.00**	0.00 ± 0.00	**0.00 ± 0.00**	0.00 ± 0.00	**0.01 ± 0.00**	0.00 ± 0.00*
	**16:0,18:3**	**608.7**	**0.01 ± 0.00**	0.01 ± 0.00	**0.07 ± 0.01**	0.05 ± 0.01	**0.03 ± 0.00**	0.04 ± 0.00	**0.35 ± 0.06**	0.16 ± 0.02
	16:1,18:2	608.7	0.00 ± 0.00	0.00 ± 0.00	0.01 ± 0.00	0.00 ± 0.00	0.02 ± 0.00	0.03 ± 0.01	0.03 ± 0.00	0.04 ± 0.01
	**16:0,18:2**	**610.5**	**0.27 ± 0.03**	0.32 ± 0.04	**1.06 ± 0.12**	0.68 ± 0.08	**0.32 ± 0.05**	0.41 ± 0.05	**2.07 ± 0.31**	0.81 ± 0.09
	**16:0,18:1**	**612.4**	**0.44 ± 0.03**	0.62 ± 0.05	**1.35 ± 0.17**	1.15 ± 0.07	**0.57 ± 0.06**	0.76 ± 0.06	**2.74 ± 0.42**	1.37 ± 0.09
	16:0,18:0	614.5	0.08 ± 0.01	0.09 ± 0.01	0.07 ± 0.01	0.05 ± 0.00	0.12 ± 0.02	0.11 ± 0.01	0.15 ± 0.02	0.06 ± 0.01
	**18:2,18:3**	**632.5**	**0.00 ± 0.00**	0.00 ± 0.00	**0.00 ± 0.00**	0.00 ± 0.00	**0.00 ± 0.00**	0.00 ± 0.00	**0.02 ± 0.00**	0.01 ± 0.00
	18:1,18:3	634.5	0.02 ± 0.00	0.03 ± 0.01	0.03 ± 0.00	0.04 ± 0.01	0.20 ± 0.02	0.22 ± 0.03	0.23 ± 0.02	0.24 ± 0.03
	**18:2,18:2**	**634.5**	**0.06 ± 0.01**	0.06 ± 0.01	**0.18 ± 0.02**	0.12 ± 0.02	**0.36 ± 0.06**	0.40 ± 0.05	**2.33 ± 0.38**	0.93 ± 0.12
	**18:1,18:2**	**636.6**	**0.38 ± 0.04**	0.35 ± 0.05	**1.37 ± 0.18**	0.91 ± 0.12	**1.10 ± 0.12**	1.21 ± 0.12	**5.56 ± 0.88**	3.52 ± 0.34
	18:0,18:2	638.5	0.30 ± 0.04	0.42 ± 0.04	0.34 ± 0.04	0.28 ± 0.02	0.05 ± 0.01	0.05 ± 0.00	0.04 ± 0.01	0.04 ± 0.00
	**18:1,18:1**	**638.5**	**1.00 ± 0.08**	1.11 ± 0.11	**3.12 ± 0.41**	3.19 ± 0.25	0.18 ± 0.02	0.17 ± 0.01	0.37 ± 0.06	0.21 ± 0.02
	18:0,18:1	640.6	0.14 ± 0.02	0.16 ± 0.01	0.21 ± 0.02	0.18 ± 0.01	0.03 ± 0.00	0.02 ± 0.00	0.02 ± 0.00	0.01 ± 0.00
	**18:0,18:0**	**642.5**	0.02 ± 0.00	0.02 ± 0.00	0.01 ± 0.00	0.01 ± 0.00	**0.00 ± 0.00**	0.00 ± 0.00	**0.01 ± 0.00**	0.00 ± 0.00*
	**16:1,22:6**	**656.6**	**0.00 ± 0.00**	0.00 ± 0.00	**0.00 ± 0.00**	0.00 ± 0.00	0.03 ± 0.00	0.03 ± 0.00	0.09 ± 0.02	0.02 ± 0.00*
	16:0,22:6	658.5	0.01 ± 0.00	0.01 ± 0.00	0.02 ± 0.00	0.01 ± 0.00	0.04 ± 0.01	0.03 ± 0.00	0.09 ± 0.02	0.02 ± 0.00*
	18:1,20:4	660.6	0.01 ± 0.00	0.01 ± 0.00	0.02 ± 0.00	0.01 ± 0.00	0.08 ± 0.01	0.09 ± 0.01	0.12 ± 0.01	0.05 ± 0.00
	**18:0,20:4**	**662.5**	0.15 ± 0.02	0.19 ± 0.02	0.05 ± 0.01	0.04 ± 0.00	**0.02 ± 0.00**	0.02 ± 0.00	**0.10 ± 0.01**	0.04 ± 0.00
	18:1,20:2	664.5	0.01 ± 0.00	0.00 ± 0.00	0.01 ± 0.00	0.00 ± 0.00	0.03 ± 0.00	0.03 ± 0.00	0.03 ± 0.01	0.02 ± 0.00
	18:2,20:1	664.5	0.03 ± 0.00	0.03 ± 0.00	0.01 ± 0.00	0.01 ± 0.00	0.02 ± 0.00	0.02 ± 0.00	0.02 ± 0.00	0.01 ± 0.00
	18:0,20:2	666.6	0.02 ± 0.00	0.02 ± 0.00	0.02 ± 0.00	0.01 ± 0.00	0.01 ± 0.00	0.01 ± 0.00	0.01 ± 0.00	0.01 ± 0.00
	18:1,20:1	666.6	0.03 ± 0.00	0.03 ± 0.00	0.02 ± 0.00	0.01 ± 0.00	0.01 ± 0.00	0.01 ± 0.00	0.02 ± 0.00	0.00 ± 0.00*
	18:1,20:0	668.5	0.01 ± 0.00	0.00 ± 0.00	0.00 ± 0.00	0.00 ± 0.00	0.02 ± 0.00	0.02 ± 0.00	0.05 ± 0.01	0.01 ± 0.00*

class	acyl chain	*m/z*	gastrocnemius				soleus			
			C	CX	D	DX	C	CX	D	DX
	18:2,22:6	682.5	0.00 ± 0.00	0.00 ± 0.00	0.00 ± 0.00	0.00 ± 0.00*	0.03 ± 0.01	0.03 ± 0.00	0.08 ± 0.02	0.03 ± 0.00*
	18:1,22:6	684.5	0.00 ± 0.00	0.00 ± 0.00	0.01 ± 0.00	0.00 ± 0.00	0.03 ± 0.00	0.03 ± 0.00	0.05 ± 0.01	0.02 ± 0.00
	18:1,22:5	686.6	0.01 ± 0.00	0.01 ± 0.00	0.02 ± 0.00	0.01 ± 0.00	0.04 ± 0.00	0.04 ± 0.00	0.03 ± 0.00	0.01 ± 0.00
	**18:1,22:4**	**688.5**	0.01 ± 0.00	0.01 ± 0.00	0.01 ± 0.00	0.01 ± 0.00	**0.00 ± 0.00**	0.00 ± 0.00	**0.02 ± 0.00**	0.01 ± 0.00
	**18:0,22:4**	**690.6**	0.01 ± 0.00	0.01 ± 0.00	0.00 ± 0.00	0.00 ± 0.00	**0.00 ± 0.00**	0.00 ± 0.00	**0.01 ± 0.00**	0.00 ± 0.00
**TAG**	**42:4**	**732.7**	0.01 ± 0.00	0.01 ± 0.00	0.00 ± 0.00	0.01 ± 0.00	**0.01 ± 0.00**	0.00 ± 0.00*	**0.10 ± 0.02**	0.04 ± 0.01
	**42:3**	**734.7**	0.00 ± 0.00	0.00 ± 0.00	0.01 ± 0.00	0.02 ± 0.00	**0.01 ± 0.00**	0.00 ± 0.00	**0.04 ± 0.00**	0.03 ± 0.00
	**42:2**	**736.7**	**0.01 ± 0.00**	0.01 ± 0.00	**0.04 ± 0.01**	0.09 ± 0.01	**0.01 ± 0.00**	0.00 ± 0.00*	**0.16 ± 0.01**	0.15 ± 0.01
	**42:1**	**738.7**	0.10 ± 0.01	0.14 ± 0.02	0.21 ± 0.04	0.41 ± 0.05	**0.08 ± 0.01**	0.03 ± 0.00	**0.59 ± 0.09**	0.37 ± 0.04
	**42:0**	**740.8**	0.04 ± 0.00	0.06 ± 0.01	0.08 ± 0.02	0.12 ± 0.02	**0.03 ± 0.00**	0.02 ± 0.00	**0.32 ± 0.09**	0.18 ± 0.01
	**44:4**	**760.7**	**0.00 ± 0.00**	0.00 ± 0.00	**0.00 ± 0.00**	0.01 ± 0.00	**0.04 ± 0.00**	0.02 ± 0.00	**0.27 ± 0.05**	0.13 ± 0.01
	**44:3**	**762.7**	**0.01 ± 0.00**	0.01 ± 0.00	**0.04 ± 0.01**	0.08 ± 0.01	11.65 ± 1.03	6.18 ± 0.80	10.38 ± 1.50	5.38 ± 0.66
	**44:2**	**764.7**	**0.05 ± 0.01**	0.05 ± 0.01	**0.21 ± 0.05**	0.44 ± 0.05	3.71 ± 0.35	1.98 ± 0.36	2.34 ± 0.39	1.13 ± 0.18
	44:1	766.7	0.13 ± 0.01	0.19 ± 0.02	0.33 ± 0.06	0.56 ± 0.06	0.71 ± 0.05	0.37 ± 0.03	1.11 ± 0.19	0.43 ± 0.05
	44:0	768.6	0.05 ± 0.01	0.06 ± 0.01	0.05 ± 0.01	0.09 ± 0.01	0.57 ± 0.04	0.25 ± 0.02	0.90 ± 0.14	0.31 ± 0.04
	**46:5**	**786.6**	0.00 ± 0.00	0.00 ± 0.00	0.00 ± 0.00	0.00 ± 0.00	**0.07 ± 0.01**	0.04 ± 0.00	**0.62 ± 0.12**	0.35 ± 0.04
	**46:4**	**788.7**	0.03 ± 0.00	0.03 ± 0.00	0.03 ± 0.01	0.05 ± 0.01	**0.08 ± 0.00**	0.05 ± 0.00	**0.41 ± 0.07**	0.23 ± 0.02
	46:3	790.8	0.09 ± 0.01	0.11 ± 0.01	0.27 ± 0.05	0.59 ± 0.08	0.06 ± 0.01	0.03 ± 0.00	0.08 ± 0.01	0.05 ± 0.01
	**46:2**	**792.7**	**0.24 ± 0.02**	0.28 ± 0.03	**0.76 ± 0.14**	1.65 ± 0.20	**1.05 ± 0.08**	0.70 ± 0.12	**7.17 ± 1.36**	4.46 ± 0.47
	**46:1**	**794.7**	0.29 ± 0.02	0.40 ± 0.04	0.49 ± 0.09	1.05 ± 0.10	**1.20 ± 0.07**	0.69 ± 0.06	**4.68 ± 0.83**	2.63 ± 0.26
	**46:0**	**796.7**	0.21 ± 0.05	0.21 ± 0.02	0.09 ± 0.02	0.18 ± 0.03	**0.71 ± 0.06**	0.37 ± 0.04	**5.31 ± 0.97**	3.01 ± 0.36
	48:5	814.7	0.01 ± 0.00	0.01 ± 0.00	0.01 ± 0.00	0.01 ± 0.00	0.01 ± 0.00	0.00 ± 0.00	0.01 ± 0.00	0.00 ± 0.00
	48:4	816.8	0.23 ± 0.02	0.29 ± 0.03	0.17 ± 0.03	0.28 ± 0.04	0.07 ± 0.01	0.04 ± 0.00	0.15 ± 0.02	0.06 ± 0.01
	**48:3**	**818.7**	0.69 ± 0.04	0.78 ± 0.08	1.77 ± 0.31	3.83 ± 0.47	**2.09 ± 0.13**	1.20 ± 0.13	**11.23 ± 2.07**	5.74 ± 0.68

class	acyl chain	*m/z*	gastrocnemius				soleus			
			C	CX	D	DX	C	CX	D	DX
	48:2	820.7	1.06 ± 0.07	1.49 ± 0.29	2.36 ± 0.45	5.55 ± 0.54	6.57 ± 0.42	3.50 ± 0.32	13.44 ± 2.46	7.27 ± 0.92
	48:1	822.8	1.42 ± 0.11	1.39 ± 0.14	1.56 ± 0.27	3.06 ± 0.32	5.96 ± 0.40	3.14 ± 0.22	10.17 ± 1.78	5.96 ± 0.72
	**48:0**	**824.8**	1.00 ± 0.05	0.46 ± 0.03	0.31 ± 0.07	0.53 ± 0.10	**0.81 ± 0.06**	0.41 ± 0.05	**4.82 ± 0.82**	2.11 ± 0.25
	50:6	840.8	0.01 ± 0.00	0.01 ± 0.00	0.01 ± 0.00	0.01 ± 0.00	0.96 ± 0.06	0.22 ± 0.01*	0.97 ± 0.21	0.51 ± 0.10
	50:5	842.7	0.06 ± 0.00	0.06 ± 0.01	0.13 ± 0.03	0.26 ± 0.04	0.02 ± 0.00	0.01 ± 0.00	0.03 ± 0.01	0.01 ± 0.00
	**50:4**	**844.7**	0.79 ± 0.05	0.86 ± 0.10	1.56 ± 0.28	2.73 ± 0.34	**0.07 ± 0.01**	0.03 ± 0.00	**0.38 ± 0.07**	0.21 ± 0.03
	50:3	846.7	2.10 ± 0.13	2.46 ± 0.26	3.73 ± 0.68	7.27 ± 0.80	1.13 ± 0.07	0.51 ± 0.04	0.64 ± 0.10	0.26 ± 0.05
	50:2	848.7	6.55 ± 0.48	7.11 ± 0.57	4.47 ± 0.76	8.81 ± 1.04	15.32 ± 0.98	8.17 ± 0.74	31.34 ± 5.75	16.96 ± 2.14
	50:1	850.7	5.90 ± 0.60	6.50 ± 0.52	3.34 ± 0.55	6.96 ± 0.81	13.91 ± 0.94	7.33 ± 0.51	23.73 ± 4.15	13.91 ± 1.68
	**50:0**	**852.7**	**1.14 ± 0.08**	0.91 ± 0.06	**0.19 ± 0.03**	0.25 ± 0.05	2.64 ± 0.15	1.19 ± 0.08	1.48 ± 0.23	0.59 ± 0.13
	52:7	866.8	0.01 ± 0.00	0.01 ± 0.00	0.01 ± 0.00	0.02 ± 0.00	0.03 ± 0.00	0.02 ± 0.00	0.09 ± 0.02	0.05 ± 0.00
	52:6	868.7	0.13 ± 0.01	0.13 ± 0.02	0.13 ± 0.02	0.20 ± 0.03	0.32 ± 0.02	0.14 ± 0.02	0.87 ± 0.14	0.36 ± 0.05
	52:5	870.7	2.11 ± 0.16	2.36 ± 0.29	1.22 ± 0.17	1.71 ± 0.23	5.12 ± 0.34	2.56 ± 0.29	8.54 ± 1.26	3.06 ± 0.38
	52:4	872.7	2.10 ± 0.20	2.32 ± 0.23	1.43 ± 0.23	2.37 ± 0.24	4.91 ± 0.33	2.54 ± 0.23	10.00 ± 1.71	4.31 ± 0.49
	52:3	874.8	8.90 ± 0.66	9.96 ± 0.86	6.04 ± 0.58	10.66 ± 1.00	21.43 ± 1.20	11.02 ± 0.90	40.07 ± 4.85	19.36 ± 2.05
	52:2	876.7	11.83 ± 1.14	14.11 ± 1.38	6.15 ± 0.66	12.96 ± 0.96	28.53 ± 1.78	15.42 ± 1.39	42.56 ± 5.22	24.40 ± 2.12
	52:1	878.7	3.44 ± 0.47	4.15 ± 0.57	7.00 ± 0.79	7.23 ± 0.85	8.43 ± 0.73	4.42 ± 0.56	8.43 ± 1.06	4.56 ± 0.59
	52:0	880.8	0.43 ± 0.04	0.33 ± 0.02	0.31 ± 0.07	0.23 ± 0.05	1.02 ± 0.10	0.43 ± 0.02	0.88 ± 0.21	0.18 ± 0.04
	**54:7**	**894.7**	**0.11 ± 0.01**	0.13 ± 0.01	**0.68 ± 0.13**	0.59 ± 0.07	**0.28 ± 0.02**	0.14 ± 0.01	**0.84 ± 0.15**	0.36 ± 0.04
	54:6	896.7	4.30 ± 0.34	4.58 ± 0.67	4.86 ± 0.53	3.04 ± 0.32	10.71 ± 0.72	4.85 ± 0.59	11.89 ± 1.58	1.77 ± 0.18*
	54:5	898.7	7.44 ± 0.87	7.67 ± 0.89	7.27 ± 0.88	5.41 ± 0.57	17.95 ± 1.39	8.27 ± 0.88	17.63 ± 2.80	3.10 ± 0.34*
	54:4	900.6	9.03 ± 0.73	9.68 ± 0.90	13.36 ± 1.32	10.51 ± 0.95	22.11 ± 1.56	10.55 ± 0.90	15.79 ± 2.15	6.12 ± 0.66
	54:3	902.7	10.83 ± 0.91	12.34 ± 1.18	26.71 ± 3.09	24.97 ± 2.32	26.56 ± 1.95	13.3 ± 1.16	31.95 ± 4.53	14.73 ± 1.46
	54:2	904.7	4.70 ± 0.57	5.92 ± 0.89	8.41 ± 1.24	8.70 ± 1.15	0.02 ± 0.00	0.01 ± 0.00	0.05 ± 0.01	0.02 ± 0.00

class	acyl chain	*m/z*	gastrocnemius				soleus			
			C	CX	D	DX	C	CX	D	DX
	54:1	906.7	1.40 ± 0.18	1.95 ± 0.41	1.81 ± 0.30	1.72 ± 0.32	0.01 ± 0.00	0.00 ± 0.00*	0.01 ± 0.00	0.00 ± 0.00*
	56:8	920.7	N.Q.	N.Q.	N.Q.	N.Q.	N.Q.	N.Q.	N.Q.	N.Q.
	**56:7**	**922.7**	**0.28 ± 0.02**	0.34 ± 0.03	**0.86 ± 0.16**	0.71 ± 0.08	0.01 ± 0.00	0.01 ± 0.00	0.04 ± 0.01	0.01 ± 0.00
	**56:6**	**924.7**	**0.22 ± 0.01**	0.23 ± 0.02	**0.73 ± 0.11**	0.51 ± 0.06	**0.03 ± 0.00**	0.01 ± 0.00	**0.22 ± 0.05**	0.13 ± 0.02
	56:5	926.8	0.19 ± 0.01	0.20 ± 0.02	0.54 ± 0.07	0.38 ± 0.04	0.48 ± 0.04	0.22 ± 0.02	0.65 ± 0.10	0.24 ± 0.03
	56:4	928.7	0.30 ± 0.03	0.34 ± 0.03	0.41 ± 0.06	0.35 ± 0.03	0.75 ± 0.06	0.37 ± 0.03	0.51 ± 0.08	0.21 ± 0.02
	56:3	930.8	0.65 ± 0.07	0.87 ± 0.06	1.56 ± 0.17	1.32 ± 0.23	1.61 ± 0.14	0.92 ± 0.07	1.89 ± 0.22	0.80 ± 0.11
	56:2	932.7	0.33 ± 0.04	0.53 ± 0.06	0.60 ± 0.08	0.51 ± 0.11	0.85 ± 0.09	0.53 ± 0.06	0.77 ± 0.12	0.32 ± 0.05
	58:10	944.7	0.10 ± 0.01	0.11 ± 0.02	0.19 ± 0.03	0.09 ± 0.01	0.25 ± 0.02	0.12 ± 0.01	0.24 ± 0.02	0.06 ± 0.01
	58:9	946.7	0.16 ± 0.01	0.19 ± 0.02	0.30 ± 0.04	0.17 ± 0.01	0.40 ± 0.03	0.20 ± 0.02	0.40 ± 0.05	0.11 ± 0.01
	58:8	948.7	0.10 ± 0.01	0.12 ± 0.01	0.17 ± 0.04	0.12 ± 0.01	0.27 ± 0.02	0.14 ± 0.01	0.23 ± 0.04	0.07 ± 0.01
	58:7	950.8	0.06 ± 0.00	0.06 ± 0.01	0.13 ± 0.02	0.10 ± 0.01	0.16 ± 0.01	0.07 ± 0.01	0.17 ± 0.02	0.06 ± 0.01
	58:6	952.8	0.05 ± 0.00	0.05 ± 0.00	0.09 ± 0.02	0.08 ± 0.01	0.13 ± 0.01	0.06 ± 0.00	0.12 ± 0.02	0.05 ± 0.01
	58:5	954.7	0.01 ± 0.00	0.01 ± 0.00	0.01 ± 0.00	0.01 ± 0.00	0.03 ± 0.00	0.01 ± 0.00	0.01 ± 0.00	0.01 ± 0.00
	58:4	956.7	0.03 ± 0.00	0.04 ± 0.01	0.06 ± 0.01	0.06 ± 0.01	0.08 ± 0.01	0.04 ± 0.00	0.08 ± 0.01	0.03 ± 0.00
	58:3	958.6	0.16 ± 0.01	0.17 ± 0.02	0.16 ± 0.03	0.18 ± 0.03	0.32 ± 0.02	0.20 ± 0.02	0.21 ± 0.03	0.11 ± 0.02
	58:2	960.7	0.09 ± 0.01	0.16 ± 0.02	0.12 ± 0.03	0.14 ± 0.02	0.23 ± 0.03	0.17 ± 0.02	0.17 ± 0.03	0.09 ± 0.01
	58:1	962.7	0.03 ± 0.01	0.07 ± 0.01	0.03 ± 0.01	0.08 ± 0.01	0.09 ± 0.01	0.09 ± 0.01	0.04 ± 0.01	0.05 ± 0.00
	60:4	984.7	0.04 ± 0.00	0.06 ± 0.01	0.04 ± 0.01	0.05 ± 0.00	0.11 ± 0.01	0.07 ± 0.01	0.05 ± 0.01	0.03 ± 0.00
	60:3	986.7	0.08 ± 0.01	0.16 ± 0.02	0.08 ± 0.02	0.10 ± 0.01	0.20 ± 0.03	0.17 ± 0.02	0.10 ± 0.02	0.06 ± 0.01
	60:2	988.8	0.05 ± 0.01	0.11 ± 0.02	0.04 ± 0.01	0.08 ± 0.01	0.13 ± 0.02	0.14 ± 0.01	0.06 ± 0.01	0.04 ± 0.00

N.Q.: not quantifiable, [a,b,c]: refers to the molecular structure of each isomer in b).

- significant change (>3-fold, p-value<0.01) between groups C and CX or D and DX marked with asterisk(*) in CX and DX column respectively.

b) PC

total chain length	acyl chains	*m/z*	total chain length	acyl chains	*m/z*	total chain length	acyl chains	*m/z*
30:0	14:0/16:0	706.5	36:2	18:0/18:2	786.5	40:7	18:2/22:5	832.6
32:2	14:0/18:2	730.6		18:1/18:1			20:3/20:4	
	16:1/16:1		36:1	16:0/20:1	788.5		22:6/18:1	
32:1	14:0/18:1	732.5		18:0/18:1		40:6	22:6/18:0	834.5
	16:0/16:1		36:0	16:0/20:0	790.6	40:5	18:0/22:5	836.5
32:0	16:0/16:0	734.5		18:0/18:0			20:1/20:4	
34:4	14:0/20:4	754.5	38:7	16:1/22:6	804.5	40:4	16:0/24:4	838.5
	16:2/18:2			18:2/20:5			18:0/22:4	
	18:3/16:1			18:3/20:4			20:1/20:3	
34:3	16:0/18:3	756.5	38:6[a]	16:0/22:6	806.5	40:1	16:0/24:1	844.5
	16:1/18:2		38:6[b]	18:2/20:4	806.6		16:1/24:0	
34:2	16:0/18:2	758.6	38:5	16:0/22:5	808.5		22:0/18:1	
34:1	16:0/18:1	760.5		18:1/20:4		40:0	16:0/24:0	846.6
34:0	18:0/16:0	762.5	38:4	16:0/22:4	810.5	42:10	22:6/20:4	854.5
36:5[a]	16:0/20:5	780.5		18:0/20:4		42:1	24:0/18:1	872.5
36:5[b]	16:1/20:4	780.6	38:3	18:0/20:3	812.5			
36:5[c]	18:3/18:2	780.5	38:2	18:0/20:2	814.6			
36:4[a]	16:0/20:4	782.5		20:0/18:2				
36:4[b]	18:2/18:2	782.5		20:1/18:1				
36:3	16:0/20:3	784.5	40:8	18:2/22:6	830.5			
	18:1/18:2			20:4/20:4				

c) PE

total chain length	acyl chains	*m/z*	total chain length	acyl chains	*m/z*	total chain length	acyl chains	*m/z*
34:3	16:1/18:2	714.5	36:2	18:0/18:2	744.5	38:3	18:0/20:3	770.6
34:2	16:0/18:2	716.5		18:1/18:1		38:2	18:0/20:2	772.5
	16:1/18:1		36:1	18:0/18:1	746.5		18:1/20:1	
34:1	16:0/18:1	718.5		20:1/16:0			20:0/18:2	
	18:0/16:1		38:7	16:1/22:6	762.5	40:8	18:2/22:6	788.6
34:0	18:0/16:0	720.6	38:6[a]	16:0/22:6	764.6	40:7	18:1/22:6	790.5
36:5	16:1/20:4	738.5	38:6[b]	18:2/20:4	764.5		22:5/18:2	
36:4[a]	16:0/20:4	740.5	38:5	16:0/22:5	766.5	40:6	22:6/18:0	792.6
36:4[b]	18:2/18:2	740.5		18:1/20:4		40:5	18:0/22:5	794.5
36:3	16:0/20:3	742.6		20:5/18:0		40:4	18:0/22:4	796.6
	18:1/18:2		38:4	16:0/22:4	768.5	40:3	18:0/22:3	798.6
				18:0/20:4				

d) TAG

total chain length	acyl chains	*m/z*	total chain length	acyl chains	*m/z*	total chain length	acyl chains	*m/z*
42:4	12:1, 12:1, 18:2	732.7	48:2	12:0, 18:1, 18:1	820.7	54:1	16:0, 18:1, 20:0	906.7
	12:1, 14:1, 16:2			14:0, 16:0, 18:2			18:0, 18:0, 18:1	
	12:1, 14:2, 16:1			14:0, 16:1, 18:1		56:8	16:0, 18:2, 22:6	920.7
	14:1, 14:1, 14:2			14:1, 16:0, 18:1			16:0, 20:4, 20:4	
42:3	12:0, 12:1, 18:2	734.7		16:0, 16:1, 16:1			18:1, 18:2, 20:5	
	12:1, 12:1, 18:1		48:1	14:0, 16:0, 18:1	822.8		18:2, 18:2, 20:4	
	12:1, 14:1, 16:1			14:0, 16:1, 18:0		56:7	16:0, 18:1, 22:6	922.7
	14:1, 14:1, 14:1			16:0, 16:0, 16:1			16:0, 18:2, 22:5	
42:2	12:0, 12:0, 18:2	736.7	48:0	16:0, 16:0, 16:0	824.8		16:0, 20:3, 20:4	
	12:0, 14:1, 16:1		50:6	14:0, 16:1, 20:5	840.8		18:1, 18:2, 20:4	
	14:0, 14:1,14:1			14:0, 18:3, 18:3			18:2, 18:2, 20:3	
42:1	12:0, 12:0, 18:1	738.7		14:1, 16:0, 20:5		56:6	16:0, 18:1, 22:5	924.7
	12:0, 14:0, 16:1			14:1, 16:1, 20:4			16:0, 18:2, 22:4	
	12:0, 14:1, 16:0			14:2, 16:0, 20:4			16:0, 20:3, 20:3	
	14:0, 14:0, 14:1			16:0, 16:3, 18:3			18:1, 18:1, 20:4	
42:0	12:0, 14:0, 16:0	740.8		16:1, 16:3, 18:2			18:1, 18:2, 20:3	
	14:0, 14:0, 14:0		50:5	14:0, 18:2, 18:3	842.7	56:5	16:0, 18:1, 22:4	926.8
44:4	12:1, 14:1, 18:2	760.7		14:1, 18:2, 18:2			16:0, 20:2, 20:3	
	12:1, 16:1, 16:2			16:1, 16:1, 18:3			18:0, 18:2, 20:3	
	14:1, 14:1, 16:2			16:1, 16:2, 18:2			18:1, 18:1, 20:3	
44:3	12:0, 14:1, 18:2	762.7	50:4	16:0, 16:1, 18:3	844.7		18:1, 18:2, 20:2	
	12:1, 14:1, 18:1			16:0, 16:2, 18:2			18:2, 18:2, 20:1	
	12:1, 16:1, 16:1			16:1, 16:1, 18:2		56:4	16:0, 20:2, 20:2	928.7
	14:1, 14:1, 16:1			16:1, 16:2, 18:1			18:1, 18:1, 20:2	
44:2	12:0, 14:0, 18:2	764.7	50:3	16:0, 16:0, 18:3	846.7		18:1, 18:2, 20:1	
	12:0, 14:1, 18:1			16:0, 16:1, 18:2		56:3	16:0, 20:1, 20:2	930.8
	12:0, 16:1, 16:1			16:1, 16:1, 18:1			18:0, 18:1, 20:2	
	14:0, 14:1, 16:1		50:2	16:0, 16:0, 18:2	848.7		18:0, 18:2, 20:1	
	14:1, 14:1, 16:0			16:0, 16:1, 18:1			18:1, 18:1, 20:1	
44:1	12:0, 14:0, 18:1	766.7	50:1	16:0, 16:0, 18:1	850.7		18:1, 18:2, 20:0	
	12:0, 16:0, 16:1		50:0	16:0, 16:0, 18:0	852.7	56:2	16:0, 20:1, 20:1	932.7
	14:0, 14:0, 16:1		52:7	14:0, 16:1, 22:6	866.8		16:1, 18:1, 22:0	
	14:0, 14:1, 16:0			14:0, 18:2, 20:5			18:0, 18:1, 20:1	
44:0	12:0, 16:0, 16:0	768.6		14:1, 16:0, 22:6			18:1, 18:1, 20:0	
	14:0, 14:0, 16:0			14:1, 18:1, 20:5		58:10	18:2, 18:2, 22:6	944.7
46:5	12:0, 14:1, 20:4	786.6		14:1, 18:2. 20:4			18:2, 20:4, 20:4	
	12:1, 16:2, 18:2			16:0, 16:2, 20:5		58:9	18:1, 18:2, 22:6	946.7
	14:1, 16:2, 16:2			16:1, 16:1, 20:5			18:1, 20:4, 20:4	
46:4	12:0, 16:1, 18:3	788.7		16:1, 16:2, 20:4			18:2, 18:2, 22:5	
	12:1, 16:1, 18:2		52:6	14:0, 18:2, 20:4	868.7	58:8	16:0, 18:2, 24:6	948.7
	14:1, 14:1, 18:2			16:1, 16:1, 20:4			18:1, 18:1, 22:6	
46:3	12:0, 16:1, 18:2	790.8		16:1, 18:2, 18:3			18:1, 18:2, 22:5	
	12:1, 16:0, 18:2		52:5	16:0, 16:0, 20:5	870.7	58:7	18:0, 18:1, 22:6	950.8

total chain length	acyl chains	*m/z*	total chain length	acyl chains	*m/z*	total chain length	acyl chains	*m/z*
	12:1, 16:1, 18:1			16:0, 16:1, 20:4			18:1, 18:1, 22:5	
	14:0, 14:1, 18:2			16:0, 18:2, 18:3		58:6	18:0, 18:1, 22:5	952.8
	14:1, 14:1, 18:1			16:1, 18:1, 18:3			18:0, 18:2, 22:4	
	14:1, 16:1, 16:1		52:4	16:0, 16:0, 20:4	872.7		18:1, 18:1, 22:4	
46:2	12:0, 16:0, 18:2	792.7		16:0, 18:1, 18:3		58:5	18:0, 18:1, 22:4	954.7
	12:0, 16:1, 18:1			16:1, 18:1, 18:2			18:0, 18:2, 22:3	
	14:0, 14:0, 18:2		52:3	16:0, 18:1, 18:2	874.8		18:0, 20:1, 20:4	
	14:0, 14:1, 18:1			16:1, 18:1, 18:1			18:1, 18:1, 22:3	
	14:0, 16:1, 16:1		52:2	16:0, 18:1, 18:1	876.7		18:1, 20:1, 20:3	
	14:1, 16:0, 16:1		52:1	16:0, 18:0, 18:1	878.7	58:4	18:1, 18:1, 22:2	956.7
46:1	12:0, 16:0, 18:1	794.7	52:0	16:0, 18:0, 18:0	880.8		18:1, 18:2, 22:1	
	14:0, 14:0, 18:1		54:7	16:1, 16:1, 22:5	894.7		18:1, 20:1, 20:2	
	14:0, 16:0, 16:1			16:1, 18:1, 20:5			18:2, 20:1, 20:1	
	14:1, 16:0, 16:0			16:1, 18:2, 20:4		58:3	16:0, 18:2, 24:1	958.6
46:0	14:0, 16:0, 16:0	796.7		18:1, 18:2, 18:4			18:0, 18:2, 22:1	
48:5	12:0, 18:2, 18:3	814.7		18:1, 18:3, 18:3			18:1, 18:1, 22:1	
	12:1, 18:2, 18:2		54:6	16:0, 16:0, 22:6	896.7		18:1, 18:2, 22:0	
	14:1, 16:1, 18:3			16:0, 18:2, 20:4			18:1, 20:1, 20:1	
	14:2, 16:1, 18:2			18:1, 18:2, 18:3		58:2	16:0, 18:1, 24:1	960.7
48:4	12:0, 18:2, 18:2	816.8		18:2, 18:2, 18:2			16:0, 20:1, 22:1	
	12:1, 18:1, 18:2		54:5	16:0, 18:1, 20:4	898.7		18:1, 18:1, 22:0	
	14:0, 16:2, 18:2			16:0, 18:2, 20:3		58:1	16:0, 18:0, 24:1	962.7
	14:1, 16:1, 18:2			18:0, 18:2, 18:3			18:0, 18:1, 22:0	
	14:1, 16:2, 18:1			18:1, 18:1, 18:3		60:4	18:1, 18:1, 24:2	984.7
	16:1, 16:1, 16:2			18:1, 18:2, 18:2			18:1, 18:2, 24:1	
48:3	12:0, 18:1, 18:2	818.7	54:4	16:0, 18:1, 20:3	900.6		18:2, 20:1, 22:1	
	14:0, 16:1, 18:2			18:0, 18:1, 18:3		60:3	18:1, 18:1, 24:1	986.7
	14:1, 16:0, 18:2			18:0, 18:2, 18:2			18:1, 18:2, 24:0	
	14:1, 16:1, 18:1			18:1, 18:1, 18:2			18:1, 20:1, 22:1	
	16:1, 16:1, 16:1		54:3	18:0, 18:1, 18:2	902.7	60:2	18:0, 18:1, 24:1	988.8
				18:1, 18:1, 18:1			18:0, 18:2, 24:0	
			54:2	16:0, 18:1, 20:1	904.7		18:1, 18:1, 24:0	
				18:0, 18:0, 18:2				
				18:0, 18:1, 18:1				

Table S3. Lipid species stimulated by diabetes (at least D/C>3-fold with p-value<0.01 in the gastrocnemius or soleus) expressed with the relative peak area of the lipid species from C (n=14), D (n = 9), DX (n = 12), and CX groups (n = 15) (number of “abundance in class” represents the percentage of peak area of each species among its corresponding head group species).

class	acyl chain	*m/z*	gastrocnemius					soleus				
			D/C	DX/C	CX/C	DX/D	% in class	D/C	DX/C	CX/C	DX/D	% in class
LPC	18:3	518.5	**3.20 ± 0.34**	4.64 ± 0.50	1.14 ± 0.13	1.45 ± 0.15	0.01	2.90 ± 0.27	1.03 ± 0.10	0.74 ± 0.07	0.35 ± 0.04	0.02
	16:1	494.5	**6.18 ± 0.59**	8.69 ± 0.79	1.17 ± 0.10	1.41 ± 0.15	0.02	**5.52 ± 0.68**	3.06 ± 0.29	0.89 ± 0.10	0.55 ± 0.06	0.05
	18:2	520.5	**4.67 ± 0.58**	5.40 ± 0.58	1.18 ± 0.13	1.16 ± 0.14	0.50	**3.91 ± 0.39**	1.80 ± 0.16	0.92 ± 0.09	0.46 ± 0.05	1.69
	20:3	546.5	**4.62 ± 0.43**	5.70 ± 0.54	0.92 ± 0.09	1.23 ± 0.12	0.06	**8.45 ± 1.16**	3.47 ± 0.44	0.93 ± 0.11	**0.41 ± 0.05****	0.10
	16:0	496.5	2.38 ± 0.25	2.09 ± 0.20	1.21 ± 0.09	0.88 ± 0.10	0.89	**4.80 ± 0.54**	2.47 ± 0.25	0.99 ± 0.10	0.52 ± 0.06**	1.56
	18:0	524.5	1.34 ± 0.16	1.39 ± 0.17	1.53 ± 0.16	1.04 ± 0.14	0.63	**4.38 ± 0.39**	2.19 ± 0.18	0.93 ± 0.08	**0.50 ± 0.04***	1.67
	20:4	544.5	2.07 ± 0.24	2.38 ± 0.24	0.90 ± 0.09	1.15 ± 0.15	0.60	**3.58 ± 0.49**	1.64 ± 0.21	0.89 ± 0.11	**0.46 ± 0.06****	0.92
	22:6	568.5	1.79 ± 0.20	1.81 ± 0.20	1.06 ± 0.15	1.01 ± 0.11	0.36	**4.12 ± 0.52**	1.60 ± 0.18	1.28 ± 0.12	**0.39 ± 0.05****	0.62
	22:5	570.5	2.81 ± 0.27	2.92 ± 0.27	1.14 ± 0.12	1.04 ± 0.09	0.11	**3.62 ± 0.40**	1.61 ± 0.18	1.03 ± 0.11	**0.45 ± 0.04****	0.19
	22:4	572.5	2.29 ± 0.23	3.29 ± 0.27	1.05 ± 0.09	1.44 ± 0.16	0.01	**3.50 ± 0.42**	1.50 ± 0.19	0.90 ± 0.11	**0.43 ± 0.06****	0.03
PC	38:2	814.5	**0.22 ± 0.04**	0.63 ± 0.11	1.37 ± 0.25	**2.87 ± 0.45****	1.22	2.00 ± 0.49	5.66 ± 1.25	5.72 ± 1.19	2.83 ± 0.48	0.18
	40:8	830.5	**0.26 ± 0.04**	0.40 ± 0.0	1.37 ± 0.17	1.53 ± 0.25*	1.79	0.42 ± 0.06	1.08 ± 0.15	2.01 ± 0.20	2.59 ± 0.41	3.99
	34:0	762.5	**0.26 ± 0.04**	0.55 ± 0.07	1.19 ± 0.15	**2.12 ± 0.34****	1.42	**0.27 ± 0.03**	0.72 ± 0.06	1.62 ± 0.17	**2.65 ± 0.31****	1.16
	36:0	790.5	**0.21 ± 0.05**	0.64 ± 0.15	1.50 ± 0.44	**2.99 ± 0.65****	0.13	**0.26 ± 0.05**	0.47 ± 0.08	1.99 ± 0.43	1.82 ± 0.29	0.63
	30:0	706.5	0.98 ± 0.16	1.76 ± 0.23	1.11 ± 0.18	1.79 ± 0.31	0.08	**0.30 ± 0.05**	0.75 ± 0.10	2.23 ± 0.26	**2.46 ± 0.39****	0.13
	42:1	872.5	0.65 ± 0.20	1.00 ± 0.21	1.14 ± 0.28	1.52 ± 0.41	0.10	**0.32 ± 0.08**	1.49 ± 0.30	3.86 ± 0.92	**4.64 ± 1.11****	0.23
LPE	16:1	452.5	**3.91 ± 0.36**	4.57 ± 0.45	1.01 ± 0.11	1.17 ± 0.10	0.05	2.16 ± 0.31	1.60 ± 0.22	1.38 ± 0.19	0.74 ± 0.09	0.07
	20:5	500.5	**4.05 ± 0.67**	3.49 ± 0.48	0.94 ± 0.16	0.86 ± 0.10	0.03	**4.20 ± 0.46**	1.25 ± 0.19	1.02 ± 0.14	**0.30 ± 0.04****	0.07
	22:5	528.5	1.34 ± 0.21	1.22 ± 0.19	0.68 ± 0.12	0.90 ± 0.12	1.76	**3.35 ± 0.39**	1.46 ± 0.19	0.56 ± 0.07	**0.44 ± 0.05****	1.87
	22:4	530.5	1.03 ± 0.14	0.98 ± 0.14	0.66 ± 0.10	0.96 ± 0.10	0.44	**3.10 ± 0.43**	2.08 ± 0.31	0.71 ± 0.11	0.67 ± 0.07	0.39
PE	34:0	720.5	**0.27 ± 0.05**	0.61 ± 0.10	1.98 ± 0.35	**2.29 ± 0.45****	0.19	0.95 ± 0.10	2.25 ± 0.28	2.56 ± 0.22	2.36 ± 0.32	0.20
	40:3	798.5	0.35 ± 0.08	0.77 ± 0.15	2.10 ± 0.48	2.18 ± 0.46	0.15	**3.38 ± 0.31**	7.66 ± 1.27	7.70 ± 0.73	**2.26 ± 0.37****	0.06
LPG	14:0	455.5	**3.44 ± 0.77**	3.94 ± 0.77	1.07 ± 0.21	1.15 ± 0.22	0.07	1.84 ± 0.28	0.50 ± 0.09	0.70 ± 0.10	0.27 ± 0.05	0.21

class	acyl chain	*m/z*	gastrocnemius					soleus				
			D/C	DX/C	CX/C	DX/D	% in class	D/C	DX/C	CX/C	DX/D	% in class
	16:1	481.5	**5.35 ± 0.91**	7.37 ± 1.31	1.32 ± 0.23	1.38 ± 0.23	0.11	**5.66 ± 0.70**	2.33 ± 0.31	1.13 ± 0.14	**0.41 ± 0.05****	0.35
PG	22:6/22:6	865.5	**0.23 ± 0.03**	0.34 ± 0.07	1.20 ± 0.17	1.49 ± 0.26	0.55	**0.29 ± 0.04**	0.28 ± 0.04	0.61 ± 0.12	0.97 ± 0.12	0.29
	16:0/16:0	721.5	0.47 ± 0.09	0.74 ± 0.14	0.87 ± 0.17	1.55 ± 0.32	2.82	**0.29 ± 0.06**	0.41 ± 0.09	0.50 ± 0.10	1.42 ± 0.34	1.26
	20:4/16:0	769.5	0.35 ± 0.06	0.40 ± 0.07	1.11 ± 0.31	1.15 ± 0.15	0.62	**0.30 ± 0.06**	0.27 ± 0.05	0.55 ± 0.15	0.88 ± 0.09	0.35
	18:2/18:0	773.5	0.40 ± 0.08	0.65 ± 0.12	1.17 ± 0.23	1.64 ± 0.34	8.52	**0.26 ± 0.06**	0.35 ± 0.07	0.65 ± 0.13	1.35 ± 0.35	3.63
LPI	18:2	595.5	**3.62 ± 0.55**	3.86 ± 0.60	1.14 ± 0.20	1.07 ± 0.18	0.64	2.98 ± 0.43	1.75 ± 0.30	0.62 ± 0.07	0.59 ± 0.10	2.73
	20:3	621.5	**4.41 ± 1.67**	4.60 ± 1.74	0.81 ± 0.30	1.04 ± 0.19	0.98	**3.56 ± 1.33**	2.07 ± 0.78	0.44 ± 0.16	0.58 ± 0.12	4.18
PI	18:2/20:4	881.5	**0.29 ± 0.04**	0.29 ± 0.05	1.02 ± 0.16	1.00 ± 0.15	0.23	0.89 ± 0.08	0.65 ± 0.07	0.65 ± 0.05	0.74 ± 0.08	0.78
LPS	18:1	522.5	**3.78 ± 0.80**	5.81 ± 1.15	1.61 ± 0.23	1.54 ± 0.36	0.62	**3.04 ± 0.79**	2.58 ± 0.62	0.67 ± 0.12	0.85 ± 0.26	2.87
PS	16:0/22:6	806.5	0.44 ± 0.11	0.54 ± 0.09	1.23 ± 0.27	1.22 ± 0.26	2.07	**0.30 ± 0.06**	0.33 ± 0.07	0.72 ± 0.17	1.12 ± 0.18	2.07
	18:0/20:4	810.5	0.56 ± 0.10	0.53 ± 0.09	1.11 ± 0.14	0.95 ± 0.19	6.93	**0.33 ± 0.07**	0.31 ± 0.07	0.67 ± 0.10	0.94 ± 0.25	6.41
	18:1/22:6	832.5	0.45 ± 0.10	0.43 ± 0.08	1.28 ± 0.32	0.94 ± 0.18	1.64	**0.32 ± 0.07**	0.29 ± 0.06	0.78 ± 0.20	0.92 ± 0.15	1.57
	18:0/22:6	834.5	0.51 ± 0.08	0.49 ± 0.07	0.90 ± 0.11	0.95 ± 0.16	54.49	**0.30 ± 0.06**	0.28 ± 0.05	0.53 ± 0.08	0.95 ± 0.23	50.83
LPA	18:2	433.5	**4.23 ± 1.40**	2.66 ± 0.70	1.36 ± 0.47	0.63 ± 0.17	7.54	1.64 ± 0.17	1.20 ± 0.13	0.95 ± 0.08	0.73 ± 0.08	23.66
PA	16:0/18:2	671.5	**3.56 ± 0.94**	2.00 ± 0.50	0.99 ± 0.13	0.56 ± 0.20	38.30	0.35 ± 0.07	0.33 ± 0.07	0.67 ± 0.09	0.92 ± 0.23	15.75
SM	d18:1/20:0	759.5	**0.31 ± 0.05**	0.41 ± 0.08	1.02 ± 0.16	1.31 ± 0.25	8.56	2.98 ± 0.24	1.29 ± 0.11	0.89 ± 0.09	0.43 ± 0.04	5.07
	d18:1/22:0	787.5	**0.26 ± 0.05**	0.38 ± 0.06	1.11 ± 0.26	1.44 ± 0.24*	12.42	0.79 ± 0.14	0.34 ± 0.06	0.82 ± 0.16	0.43 ± 0.07	3.03
	d18:1/23:0	801.5	**0.16 ± 0.04**	0.24 ± 0.06	0.83 ± 0.18	1.50 ± 0.34*	6.86	0.49 ± 0.09	0.20 ± 0.04	0.62 ± 0.11	0.41 ± 0.08	1.79
	d18:1/24:2	811.5	**0.22 ± 0.04**	0.34 ± 0.06	1.04 ± 0.30	1.50 ± 0.18*	5.91	0.67 ± 0.14	0.30 ± 0.06	0.77 ± 0.20	0.45 ± 0.05	1.44
	d18:1/24:0	815.5	**0.21 ± 0.04**	0.35 ± 0.06	1.04 ± 0.19	1.68 ± 0.34*	18.42	0.61 ± 0.12	0.30 ± 0.06	0.77 ± 0.13	0.49 ± 0.09	54.02
Cer	d18:1/24:1	648.5	**0.33 ± 0.06**	0.52 ± 0.09	1.21 ± 0.21	1.56 ± 0.24	26.15	1.18 ± 0.20	0.48 ± 0.07	0.99 ± 0.15	0.41 ± 0.06	24.21
	d18:1/24:0	650.5	**0.22 ± 0.04**	0.45 ± 0.09	1.14 ± 0.20	**2.04 ± 0.39****	42.31	0.74 ± 0.11	0.41 ± 0.07	0.92 ± 0.14	0.56 ± 0.08	40.55
	d18:1/16:0	538.5	1.31 ± 0.24	1.45 ± 0.24	1.03 ± 0.16	1.10 ± 0.22	0.78	**3.43 ± 0.33**	1.02 ± 0.08	0.92 ± 0.07	**0.30 ± 0.03****	1.92
	d18:0/18:1	564.5	0.83 ± 0.13	0.79 ± 0.13	1.11 ± 0.18	0.95 ± 0.17	0.12	**3.12 ± 0.14**	0.90 ± 0.04	0.95 ± 0.05	**0.29 ± 0.01****	1.30
DAG	18:1,18:1	638.5	**3.13 ± 0.48**	3.20 ± 0.36	1.11 ± 0.15	1.02 ± 0.15	30.76	2.10 ± 0.39	1.17 ± 0.16	0.95 ± 0.13	0.55 ± 0.09	4.89

class	acyl chain	*m/z*	gastrocnemius					soleus				
			D/C	DX/C	CX/C	DX/D	% in class	D/C	DX/C	CX/C	DX/D	% in class
	16:1,22:6	656.5	**5.87 ± 1.59**	2.96 ± 0.76	0.97 ± 0.26	0.50 ± 0.14	0.00	2.92 ± 0.70	0.80 ± 0.15	0.90 ± 0.15	0.28 ± 0.07	0.85
	14:1,16:0	556.5	**7.04 ± 1.48**	4.46 ± 0.86	1.02 ± 0.18	0.63 ± 0.14	0.01	**3.92 ± 0.87**	1.79 ± 0.36	1.25 ± 0.21	**0.46 ± 0.11****	0.28
	16:1,18:3	606.5	**8.84 ± 2.23**	3.99 ± 0.76	0.99 ± 0.20	0.45 ± 0.12	0.01	**7.97 ± 1.77**	2.27 ± 0.33	1.00 ± 0.11	**0.28 ± 0.07****	0.03
	16:0,18:3	608.5	**7.20 ± 1.41**	5.33 ± 1.08	1.14 ± 0.21	0.74 ± 0.16	0.29	**12.4 ± 2.93**	5.80 ± 1.21	1.41 ± 0.28	**0.47 ± 0.10***	0.79
	16:0,18:2	610.5	**3.87 ± 0.61**	2.50 ± 0.39	1.16 ± 0.20	0.65 ± 0.10	8.43	**6.50 ± 1.43**	2.53 ± 0.49	1.28 ± 0.26	**0.39 ± 0.07****	8.83
	16:0,18:1	612.5	**3.08 ± 0.45**	2.62 ± 0.25	1.40 ± 0.16	0.85 ± 0.12	13.56	**4.83 ± 0.89**	2.41 ± 0.29	1.34 ± 0.17	**0.50 ± 0.08****	15.76
	18:2,18:3	632.5	**4.93 ± 1.14**	3.24 ± 0.64	1.06 ± 0.21	0.66 ± 0.17	0.02	**7.02 ± 1.63**	2.54 ± 0.52	1.10 ± 0.20	**0.36 ± 0.09***	0.06
	18:2,18:2	634.5	**3.05 ± 0.54**	1.93 ± 0.36	0.98 ± 0.17	0.63 ± 0.12	1.85	**6.54 ± 1.54**	2.62 ± 0.55	1.13 ± 0.24	**0.40 ± 0.08****	9.90
	18:1,18:2	636.5	**3.64 ± 0.62**	2.40 ± 0.42	0.94 ± 0.16	0.66 ± 0.12	11.63	**5.06 ± 0.96**	3.20 ± 0.46	1.10 ± 0.16	0.63 ± 0.12	30.50
	14:0,16:0	558.5	2.39 ± 0.45	1.29 ± 0.23	1.12 ± 0.20	0.54 ± 0.10	0.11	**3.87 ± 0.98**	1.72 ± 0.41	1.12 ± 0.24	**0.44 ± 0.11****	0.17
	18:0,18:0	642.5	0.62 ± 0.09	0.48 ± 0.15	1.03 ± 0.14	0.77 ± 0.23	0.59	**5.52 ± 1.18**	1.71 ± 0.32	0.93 ± 0.13	**0.31 ± 0.08****	0.03
	18:0,20:4	662.5	0.36 ± 0.06	0.24 ± 0.03	1.27 ± 0.21	0.67 ± 0.10	4.61	**4.45 ± 0.82**	1.68 ± 0.28	1.12 ± 0.17	**0.38 ± 0.07***	0.61
	18:1,22:4	688.5	1.05 ± 0.19	0.59 ± 0.08	0.88 ± 0.13	0.56 ± 0.10	0.27	**9.61 ± 2.02**	3.73 ± 0.65	1.16 ± 0.16	**0.39 ± 0.09****	0.05
	18:0,22:4	690.5	0.35 ± 0.07	0.23 ± 0.03	1.18 ± 0.18	0.67 ± 0.12	0.33	**3.55 ± 0.77**	1.20 ± 0.23	1.19 ± 0.21	**0.34 ± 0.06****	0.09
TAG	44:3	762.7	**4.46 ± 1.18**	8.14 ± 1.41	1.32 ± 0.33	1.82 ± 0.47	0.01	0.89 ± 0.15	0.46 ± 0.07	0.53 ± 0.08	0.52 ± 0.10	5.22
	44:2	764.7	**4.53 ± 1.17**	9.38 ± 1.65	1.09 ± 0.20	**2.07 ± 0.51***	0.05	0.63 ± 0.12	0.31 ± 0.06	0.53 ± 0.11	0.48 ± 0.11	1.66
	50:0	852.7	**0.17 ± 0.03**	0.22 ± 0.05	0.80 ± 0.07	1.34 ± 0.33	1.24	0.56 ± 0.09	0.22 ± 0.05	0.45 ± 0.04	0.40 ± 0.10	1.18
	56:7	922.7	**3.03 ± 0.59**	2.49 ± 0.34	1.20 ± 0.14	0.82 ± 0.18	0.31	2.97 ± 0.53	1.00 ± 0.12	0.44 ± 0.04	0.34 ± 0.06	0.01
	42:2	736.7	**5.25 ± 1.35**	11.02 ± 2.02	1.05 ± 0.18	**2.10 ± 0.50***	0.01	**11.47 ± 2.07**	11.08 ± 2.12	0.30 ± 0.06	0.97 ± 0.09	0.01
	44:4	760.7	**4.00 ± 1.19**	6.76 ± 1.40	1.21 ± 0.23	1.69 ± 0.47	0.00	**6.53 ± 1.21**	3.04 ± 0.40	0.60 ± 0.06	**0.47 ± 0.10***	0.02
	46:2	792.7	**3.19 ± 0.64**	6.87 ± 1.03	1.16 ± 0.17	**2.15 ± 0.46***	0.26	**6.80 ± 1.39**	4.23 ± 0.56	0.66 ± 0.12	0.62 ± 0.14*	0.47
	54:7	894.7	**6.12 ± 1.22**	5.30 ± 0.76	1.19 ± 0.16	0.87 ± 0.20	0.12	**3.05 ± 0.59**	1.30 ± 0.16	0.52 ± 0.06	**0.43 ± 0.09****	0.12
	56:6	924.7	**3.26 ± 0.50**	2.29 ± 0.31	1.00 ± 0.10	0.70 ± 0.13	0.24	**7.36 ± 1.63**	4.27 ± 0.63	0.49 ± 0.06	0.58 ± 0.14*	0.01
	42:4	732.7	0.60 ± 0.14	1.01 ± 0.18	1.13 ± 0.18	1.68 ± 0.35	0.01	**7.20 ± 1.84**	2.61 ± 0.66	0.20 ± 0.04	**0.36 ± 0.11***	0.01
	42:3	734.7	2.31 ± 0.56	4.79 ± 0.87	1.15 ± 0.21	2.08 ± 0.47	0.00	**6.30 ± 1.60**	5.33 ± 1.31	0.38 ± 0.09	0.85 ± 0.11	0.00
	42:1	738.7	2.07 ± 0.45	4.09 ± 0.65	1.36 ± 0.22	1.98 ± 0.45	0.11	**7.55 ± 1.59**	4.66 ± 0.89	0.42 ± 0.08	0.62 ± 0.12	0.04

class	acyl chain	*m/z*	gastrocnemius					soleus				
			D/C	DX/C	CX/C	DX/D	% in class	D/C	DX/C	CX/C	DX/D	% in class
	42:0	740.7	1.85 ± 0.43	2.64 ± 0.46	1.24 ± 0.20	1.43 ± 0.35	0.05	**9.47 ± 2.99**	5.29 ± 0.69	0.52 ± 0.07	0.56 ± 0.17	0.02
	46:5	786.7	1.06 ± 0.26	1.36 ± 0.24	1.13 ± 0.17	1.28 ± 0.34	0.00	**8.75 ± 1.84**	5.00 ± 0.78	0.52 ± 0.08	0.57 ± 0.13**	0.03
	46:4	788.7	1.31 ± 0.29	1.78 ± 0.27	1.15 ± 0.14	1.37 ± 0.32	0.03	**4.90 ± 0.90**	2.83 ± 0.33	0.64 ± 0.07	0.58 ± 0.12**	0.04
	46:1	794.7	1.69 ± 0.32	3.59 ± 0.43	1.37 ± 0.16	2.13 ± 0.43	0.32	**3.89 ± 0.73**	2.19 ± 0.25	0.57 ± 0.06	0.56 ± 0.11**	0.54
	46:0	796.7	0.42 ± 0.12	0.86 ± 0.23	0.96 ± 0.23	2.06 ± 0.54	0.23	**7.49 ± 1.53**	4.24 ± 0.64	0.52 ± 0.07	0.57 ± 0.12**	0.32
	48:3	818.7	2.57 ± 0.48	5.55 ± 0.77	1.13 ± 0.14	2.16 ± 0.47	0.75	**5.38 ± 1.05**	2.75 ± 0.37	0.57 ± 0.07	0.51 ± 0.11**	0.94
	48:0	824.7	0.31 ± 0.07	0.53 ± 0.10	0.46 ± 0.03	1.74 ± 0.50	1.09	**5.96 ± 1.12**	2.61 ± 0.37	0.51 ± 0.07	**0.44 ± 0.09****	0.36
	50:4	844.7	1.97 ± 0.38	3.46 ± 0.47	1.09 ± 0.15	1.76 ± 0.39	0.86	**5.68 ± 1.18**	3.07 ± 0.48	0.42 ± 0.06	0.54 ± 0.13*	0.03

- species marked as **bold** in D/C column represent significant change (>3-fold, p-value < 0.01 in D/C)
- species marked as **bold** in DX/D column represent significant change (>3-fold, p-value < 0.01 in D/C and >2-fold, p-value < 0.05 in DX/D)
- species written in <u>underline</u> denote high abundance in each class from group C
- * p<0.05, ** p<0.01 : significant change between groups D and DX marked in DX/D column

Table S4. Training intensity of rats on a treadmill.

Age (weeks old)	Intensity (m/min)	Time (min)
8	10	10
9	12	25
10	11–12.5	35
11	12.5–14.5	40
12	14.5	40
13	14.5	40
14	14.5	50

Table S5. SRM transitions for quantifying lipids species according to the class

**Class**	**Precursor ion**	**Product ion**	**Collision energy (V)**
LPC	$[M+H]^+$	[a]$[Pcho+H]^+$	40
PC	$[M+H]^+$	[a]$[Pcho+H]^+$	40
LPE	$[M+H]^+$	[b]$[M+H-141]^+$	20
PE	$[M+H]^+$	[b]$[M+H-141]^+$	20
LPG	$[M-H]^-$	$[RCOO]^-$	35
PG	$[M-H]^-$	$[RCOO]^-$	35
LPI	$[M-H]^-$	$[RCOO]^-$	35
PI	$[M-H]^-$	$[RCOO]^-$	35
LPS	$[M-H]^-$	$[M-H-RCOOH]^-$	35
PS	$[M-H]^-$	$[RCOO]^-$	35
LPA	$[M-H]^-$	$[M-H-RCOOH]^-$	35
PA	$[M-H]^-$	$[RCOO]^-$	35
SM	$[M+H]^+$	[a]$[Pcho+H]^+$	40
Cer	$[M+H]^+$	[c]$[d18:1]^+$	30
MHC	$[M+H]^+$	[c]$[d18:1]^+$	30
DAG	$[M+NH_4]^+$	$[M+NH_4-RCOONH_4]^+$	25
TAG	$[M+NH_4]^+$	$[M+NH_4-RCOONH_4]^+$	25

[a]protonated phosphocholine ion, [b]loss of phosphoethanolamine, [c]sphingosine long chain base fragments
